# A Laminating Strategy to Manyfold Enhance the Elastic Stretchability of Stretchable Electronics

**DOI:** 10.1002/advs.202521763

**Published:** 2026-01-25

**Authors:** Zanxin Zhou, Xiaolei Wu, Xinkai Xu, Fanming Wang, Shuang Li, Huiling Li, Yewang Su

**Affiliations:** ^1^ State Key Laboratory of Nonlinear Mechanics Institute of Mechanics Chinese Academy of Sciences Beijing China; ^2^ School of Engineering Science University of Chinese Academy of Sciences Beijing China; ^3^ Department of Bioengineering University of California Los Angeles California USA; ^4^ Institute of Biomechanics and Medical Engineering Applied Mechanics Laboratory Department of Engineering Mechanics Tsinghua University Beijing China; ^5^ Zhongke Technology Achievement Transfer and Transformation Center of Henan Province Changyuan County Henan China

**Keywords:** elastic stretchability, laminating strategy, thin‐ribbon stretchable electronics

## Abstract

Achieving high elastic stretchability remains a central focus and persistent challenge in stretchable electronics. Widely adopted fabrication techniques like coating and photolithography produce thin‐ribbon metallic structures that facilitate stretchability through various strategies. Nevertheless, their ultra‐thin nature causes out‐of‐plane buckling and stress concentration, limiting elastic stretchability, especially on substrates with elastic moduli in the megapascal range, greatly restricting practical use. Here, we propose a laminating strategy that laminates a thick‐polymer layer onto the thin‐ribbon metallic structure. Using serpentine structures on soft and hard substrates as representative cases, this approach increases elastic stretchability by 3.5‐fold and 2.3‐fold. The enhancement results from transforming the deformation mode from out‐of‐plane buckling to in‐plane bending, effectively reducing stress concentration and generating significant angles for straight segments to endure larger stretching. As a device‐level example, this strategy enables stretchable sensors to measure large strains in smart tires with hard substrates and shows broad potential in thin‐ribbon electronics.

## Graphical abstract

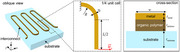

## 1 Introduction

Stretchable electronics, owing to their inherent flexibility and capability for conformal integration with complex surfaces, have found important applications across a broad spectrum of fields [1, 2, 3], spanning healthcare, aerospace, and industrial manufacturing. They enable real‐time physiological monitoring and disease diagnosis [4, 5, 6, 7, 8, 9], facilitate strain sensing on aircraft surfaces and adaptive smart skins [10, 11, 12, 13], and are employed in soft robotics and for pipeline and pressure vessel health monitoring [14, 15, 16]. These cross‐disciplinary applications critically rely on the ability of devices to maintain stable performance under deformation [17, 18, 19, 20], with high elastic stretchability being a key requirement. To achieve or enhance this property, a variety of structural designs and material engineering strategies have been proposed, but preserving excellent electrical stability under large strains remains a persistent challenge [21, 22, 23] that continues to attract significant attention from the research community.

**FIGURE 1:**
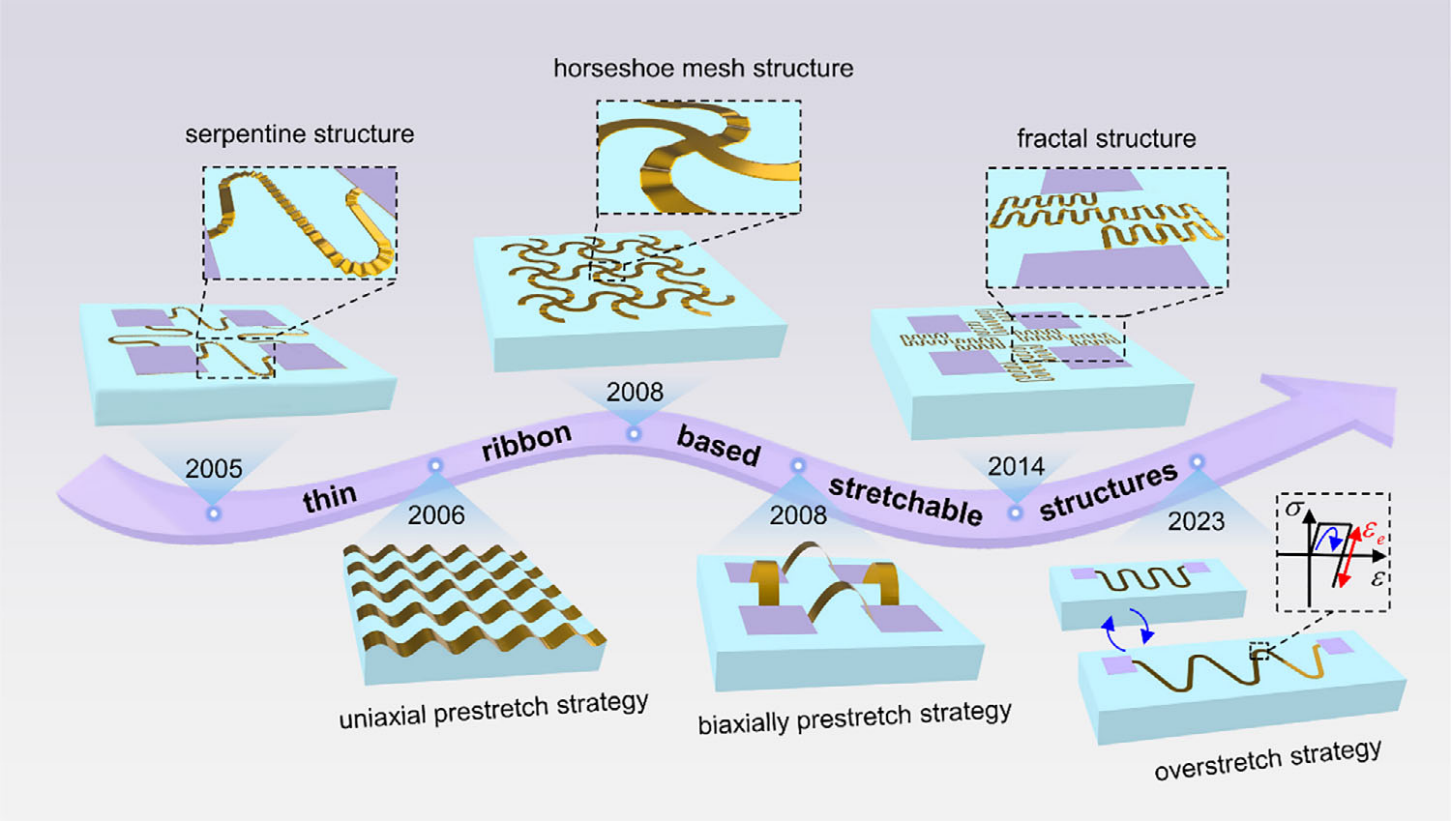
Evolution of stretchable structures over the past decades.

As shown in Figure 1, based on conventional and widely adopted fabrication techniques such as coating and photolithography [24, 25, 26], various thin‐ribbon metallic structures, approximately 1 µm or even thinner, have been developed to enable elastic stretchability in stretchable electronics. 1) **By designing geometric layouts**. The advent of versatile stretchable structures, including serpentine [27], horseshoe mesh [28], and fractal [29] structures, confers inherent elastic compliance to the devices. 2) **By using the prestrained elastic substrate**. The thin‐ribbon structure is transferred onto a prestrained elastic substrate and subsequently released [30, 31, 32], forming a 3D architecture capable of accommodating larger deformations. 3) **Overstretch strategy**. Our group has developed a distinctive overstretch strategy [33] in which the thin‐ribbon structure is first bonded to an elastic substrate, then subjected to controlled deformation beyond its elastic limit before strain release, effectively doubling the elastic stretchability of the device. Nevertheless, the ultra‐thin nature of these structures makes them susceptible to out‐of‐plane buckling and stress concentration during stretching, particularly on substrates with elastic moduli in the megapascal range, restricting their operation to a narrowly defined stretching range. Owing to this constraint, the purely elastic stretchability of these structures remains insufficient to meet the requirements in scenarios involving large deformations. Therefore, proposing ingenious strategies to enhance their purely elastic stretchability remains a critical and enduring theme.

In this paper, a laminating strategy is proposed to manyfold enhance the elastic stretchability of stretchable electronics. Using serpentine structures on soft and hard substrates as representative cases, this approach increases elastic stretchability by 3.5‐fold and 2.3‐fold. The theoretical, numerical, and experimental results collectively prove that the strategy by laminating a thick‐polymer layer to the thin‐ribbon metallic structure induces a transformation of the deformation mode from out‐of‐plane buckling to in‐plane bending, which effectively reduces stress concentration. Furthermore, the bending of the arc generates significant angles in the straight segments, resulting in a substantial improvement in elastic stretchability. The combination of the laminating and the overstretch strategy yields a synergistic enhancement effect applicable to stretchable structures with various geometries. Moreover, the laminating strategy overcomes the constraints imposed by hard elastic substrates on the elastic stretchability, enabling stretchable sensors to measure local large strains in smart tires with high elastic modulus. The laminating strategy offers a fundamental guidance for the design and fabrication of stretchable electronic based on extensively adopted fabrication technologies.

## 2 Results

### 2.1 Schematic Diagram and Mechanism of the Laminating Strategy

**FIGURE 2:**
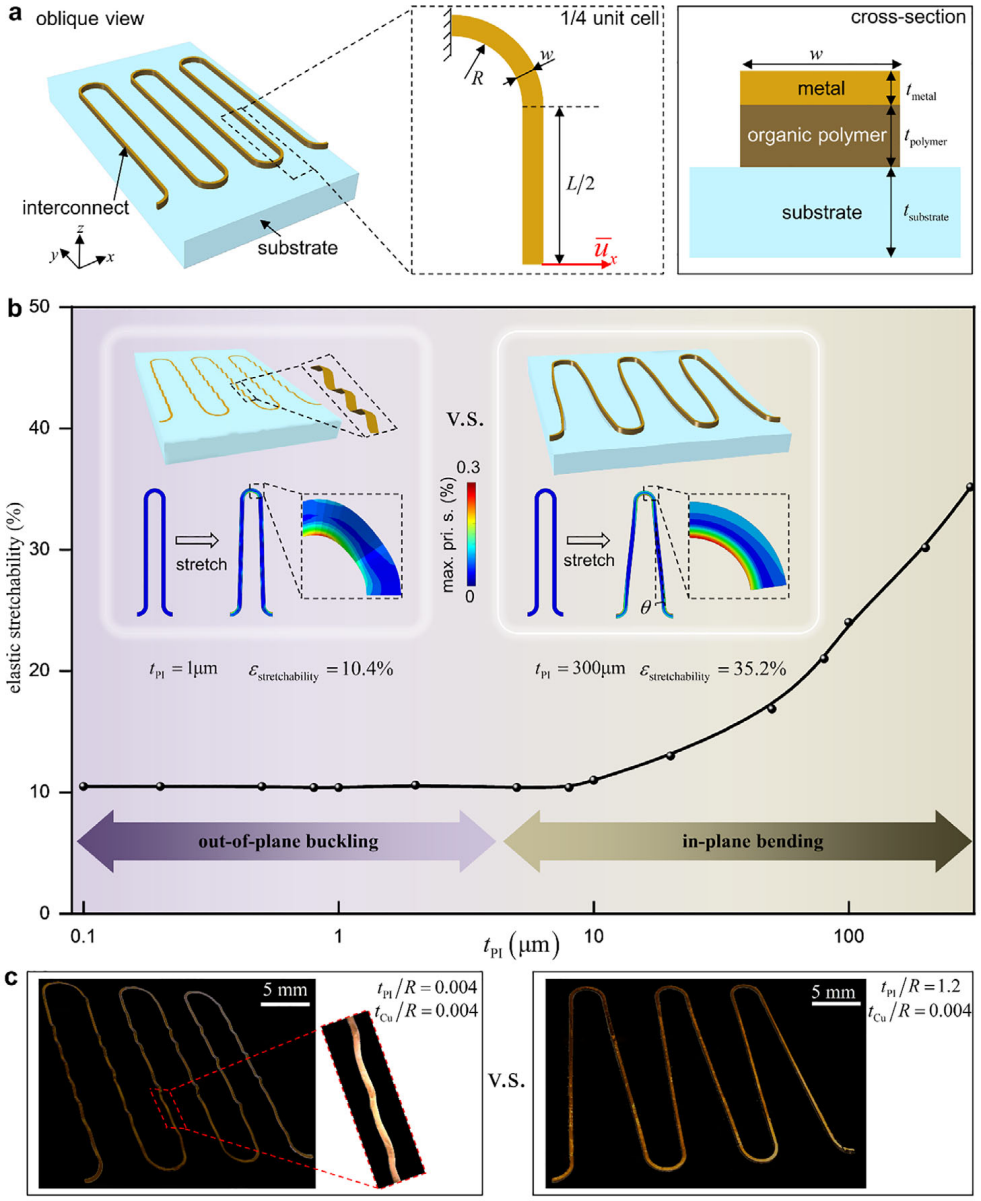
Display and Mechanism of the Laminating Strategy: a) Schematic diagram of the laminating strategy model. b) Effect of PI thickness on elastic stretchability and its mechanism analysis. c) Experimental validation of the laminating strategy mechanism.

A common feature of stretchable electronic fabricated via conventional fabrication technologies of vapor deposition and photolithography typically is the ultra‐thin geometry structure. However, this geometric feature leads to pronounced out‐of‐plane buckling during stretching, resulting in localized strain concentration that severely limits the elastic stretchability. Therefore, the laminating strategy compatible with the conventional fabrication technologies is developed: as shown in Figure 2a, by laminating a thick organic polymer layer with thickness *t*_polymer_ onto the ultrathin metal layer with thickness *t*_metal_, followed by bonding the composite interconnect to the elastic substrate with thickness *t*_sub_, this configuration transforms the deformation mode of stretchable electronics from out‐of‐plane buckling to in‐plane bending, effectively resolving the aforementioned challenges. The serpentine interconnect consists of periodic cells, each containing two straight segments with length *L* and two circular arcs with radius *R*, maintaining a constant conductive trace width *w* throughout the structure.

The FEA (see the **Methods** section) is performed with a focus on the elastic stretchability to investigate the strategy, defined as the maximum applied strain before plastic deformation in Cu material of the interconnect. In FEA, the 45‐µm‐wide interconnect with *R* = 0.25mmand *L* = 3mm consists of a 1‐µm‐thick copper (Cu) layer with *E*_Cu_ = 127GPa and σ_s_ = 372MPa and a variable thickness polyimide (PI) layer with *E*_PI_ = 70GPa bonded to 0.5‐mm‐thick Ecoflex substrate with*E*_sub_ = 60kPa. As shown in Figure 2b, the elastic stretchability exhibits two regimes (Notably, the actual FEA results indicate that there is no abrupt demarcation point between the two deformation modes, but rather a continuous and gradual transition process. To facilitate systematic analysis and discussion, 10 µm is selected as a representative thickness example to illustrate the dominant roles of different deformation mechanisms): 1) **The out‐of‐plane buckling regime**. When *t*_PI_ < 10µm, the elastic stretchability remains nearly independent of *t*_PI_. Taking the 1‐µm‐thick PI as an example, the interconnect demonstrates significant out‐of‐plane buckling, with predominant waved deformation along the out‐of‐plane direction and minimal deformation in the in‐plane direction, which is further experimentally confirmed in Figure 2c for interconnects with *t*_PI_/*R* = 0.004. Thus, the elastic stretchability of the interconnect is constrained to 10.4%. To further analyze the phenomenon, this study calculates the effective bending stiffness ${\bar{EI}}_{\mathrm{out}-\mathrm{of}-\mathrm{plane}}$ and ${\bar{EI}}_{\mathrm{in}-\mathrm{plane}}$, which governs the out‐of‐plane and in‐plane deformation of the interconnect, respectively (see Note S1). Due to *E*_PI_ ≪ *E*_Cu_, ${\bar{EI}}_{\mathrm{in}-\mathrm{plane}}$ and ${\bar{EI}}_{\mathrm{out}-\mathrm{of}-\mathrm{plane}}$ show little dependence on *t*_PI_ and ${\bar{EI}}_{\mathrm{in}-\mathrm{plane}}>{\bar{EI}}_{\mathrm{out}-\mathrm{of}-\mathrm{plane}}$ (see Figure S1). Consequently, during this regime, the interconnect exhibits the out‐of‐plane buckling mode while retaining its elastic stretchability. 2) **The in‐plane bending regime**. When *t*_PI_ > 10µm, the elastic stretchability improves significantly with increasing *t*_PI_. For the 300‐µm‐thick PI, the interconnect primarily undergoes in‐plane deformation without out‐of‐buckling, which can be further experimentally observed in the interconnect composed of thick PI with *t*_PI_/*R* = 1.2. Further observation reveals that, the in‐plane bending of the arc segments induces a significant angle θ in the straight segments, dominating the overall deformation of the interconnect and enhancing the elastic stretchability to 35.2%. The reasons are as follows: during this regime, ${\bar{EI}}_{\mathrm{out}-\mathrm{of}-\mathrm{plane}}$ rises rapidly with increasing *t*_PI_, while ${\bar{EI}}_{\mathrm{out}-\mathrm{of}-\mathrm{plane}}>>{\bar{EI}}_{\mathrm{in}-\mathrm{plane}}$(Figure S1a). Consequently, the deformation mode of the interconnect transitions from out‐of‐plane buckling to in‐plane bending with increasing *t*_PI_. Figure S2 further demonstrates the transition of the deformation mode of the interconnect through schematic diagrams of FEA at *t*_PI_ = 1, 2, 10, 20, 100, 200µm.

To theoretically analyze the limit of the elastic stretchability of the serpentine interconnect based on the laminating strategy, a quarter‐unit‐cell analytical model is established, which is treated as the Euler‐Bernoulli beam, as shown in Figure S3c. Based on the model, the limit ε_limit of interconnect_ can be obtained as (details shown in Note S2):

(1) $$\varepsilon_{\mathrm{limit}\;\mathrm{of}\;\mathrm{interconnect}}=2\bar{R}\left(\frac{{\bar{L}}^{3}}{12\bar{L}+24}+\frac{\pi {\bar{L}}^{2}+8\bar{L}+2\pi }{4\bar{L}+8}\right)\varepsilon_{\mathrm{limit}\;\mathrm{of}\;\mathrm{metal}}$$

here, $\bar{L}=L/R$ and $\bar{R}=R/w$ are the dimensionless geometric parameters, ε_limit of metal_ represents the limit of metal elasticity. For Cu with ε_limit of metal_ = 0.3%, the ε_limit of interconnect_ reaches 76.8%. To validate the result, the range of *t*_PI_ is extended to 1 − 10000µmin FEA. Figure S3d demonstrates that in the in‐plane bending regime, the elastic stretchability initially improves with increasing *t*_PI_, then tends to be stable after *t*_PI_ exceeds 5000µm. Excellent agreement between the theoretical results from Equation 1 and FEA results in the stable regime confirms the validity of the analytical model. However, due to the dimensional constraints in practical applications, the investigation is primarily focused on configurations with a thickness of PI below that of the elastic substrate, where *t*_PI_ ≤ 300µm.

**FIGURE 3:**
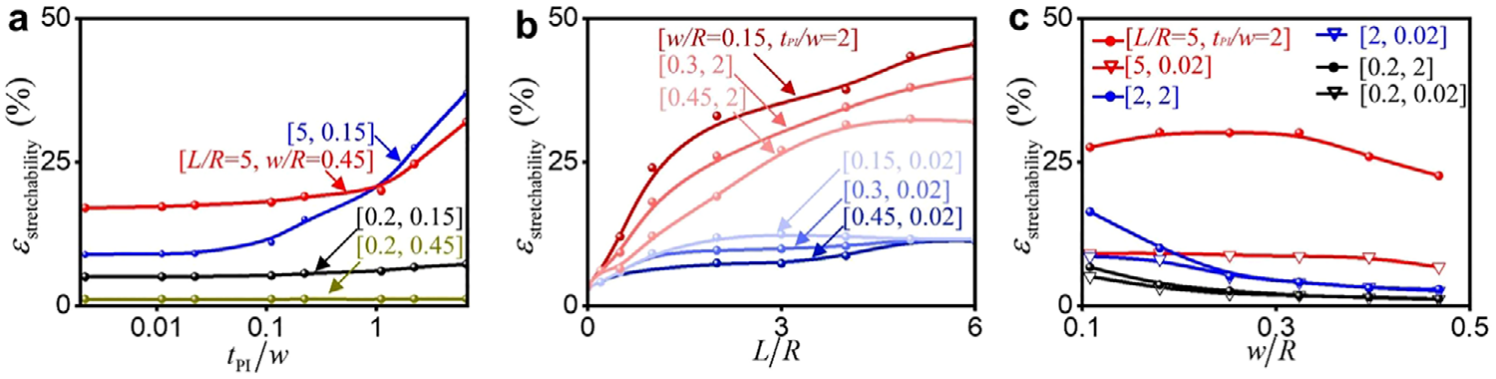
Effect of Geometric Parameters on Lamination Strategy: a) The influence of geometric parameters on the laminating strategy. b) The effect of *L/R* on the elastic stretchability of structures with and without the laminating strategy. c) The effect of *w/R* on the elastic stretchability of structures with and without the laminating strategy.

To offer practical guidance, this study analyzes the influence of geometric parameters on laminating strategy. Figure 3a and S4 present the effect of dimensionless length *L/R* and width *w/R* on the feasibility of the laminating strategy. The comparisons between curves [*L/R* = 5, *w/R* = 0.45] and [*L/R* = 0.2, *w/R* = 0.45], as well as curves [*L/R* = 5, *w/R* = 0.15] and [*L/R* = 0.2, *w/R* = 0.15], demonstrates that the laminating strategy can significantly enhance the elastic stretchability of interconnects with larger *L/R*, which indicates that *L/R* is critical for the application of this strategy. The elastic stretchability exhibits consistent variation trends for the interconnects with different *w/R*, confirming the negligible effect of *w/R* on the feasibility of this strategy (comparing curves [*L/R* = 5, *w/R* = 0.45] and [*L/R* = 5, *w/R* = 0.15], as well as curves [*L/R* = 0.2, *w/R* = 0.45] and [*L/R* = 0.2, *w/R* = 0.15]). Furthermore, this study analyzes the influence of *L/R* and *w/R* on the enhancement of the laminating strategy. As shown in Figure 3b,c, the disparity in the elastic stretchability progressively improves with increasing *L/R* and decreases with increasing *w/R* between interconnects with different *t*_PI_ */w*, demonstrating that greater *L/R* and lower *w/R* can more effectively improve the enhancement of laminating strategy. However, the elastic stretchability exhibits minor discrepancies in curves [*L/R* = 0.2, *t*_PI_ */w* = 0.02] and [*L/R* = 0.2, *t*_PI_ */w* = 2] of Figure 3c, as the laminating strategy is inherently unsuitable for interconnects with small *L/R*. In summary, a structural design featuring larger dimensionless length and smaller dimensionless width is recommended, when implementing the laminating strategy.

Polymer mechanical properties, as a key factor influencing the bending stiffness and torsional stiffness of interconnects, affects their deformation modes and the enhancement effectiveness of the laminating strategy. Based on FEA, the influence of the polymer mechanical properties on the laminating strategy is investigated. Varying 300‐µm‐thick polymer layers (including PI, PP, PET, PTFE, CE) with different mechanical properties are laminated onto metal structures (Cu) of 1 µm and calculated the relationship between the elastic stretchability of the structure and the elastic modulus (see Table S2). As shown in Figure S17, as the elastic modulus of the polymer material increases, the elastic stretchability of the laminated structure decreases. This study provides guidance for the material selection in the laminating strategy.

And Metal thickness, as a key factor influencing the mechanical parameters of interconnects, affects their deformation modes and the enhancement effectiveness of the laminating strategy. Based on FEA, the influence of metal thickness on the laminating strategy is investigated. A thick PI layer (300 µm) is laminated onto metal structures (Cu) of varying thicknesses and calculated the relationship between the elastic stretchability of the structure and the metal thickness. As shown in Figure S18, the elastic stretchability initially increases with increasing metal thickness and then stabilizes. This is because increasing the metal thickness can achieve a similar effect as increasing the polymer thickness, leading to an initial rise and subsequent stabilization in the elastic stretchability of the interconnect structure. Although the thick metal layer can similarly enhance the elastic stretchability of the structure, most metal layers in practice remain thin‐ribbon structures due to fabrication constraints, thereby limiting their applicability.

### 2.2 Combination of the Laminating Strategy and Overstretch Strategy

**FIGURE 4:**
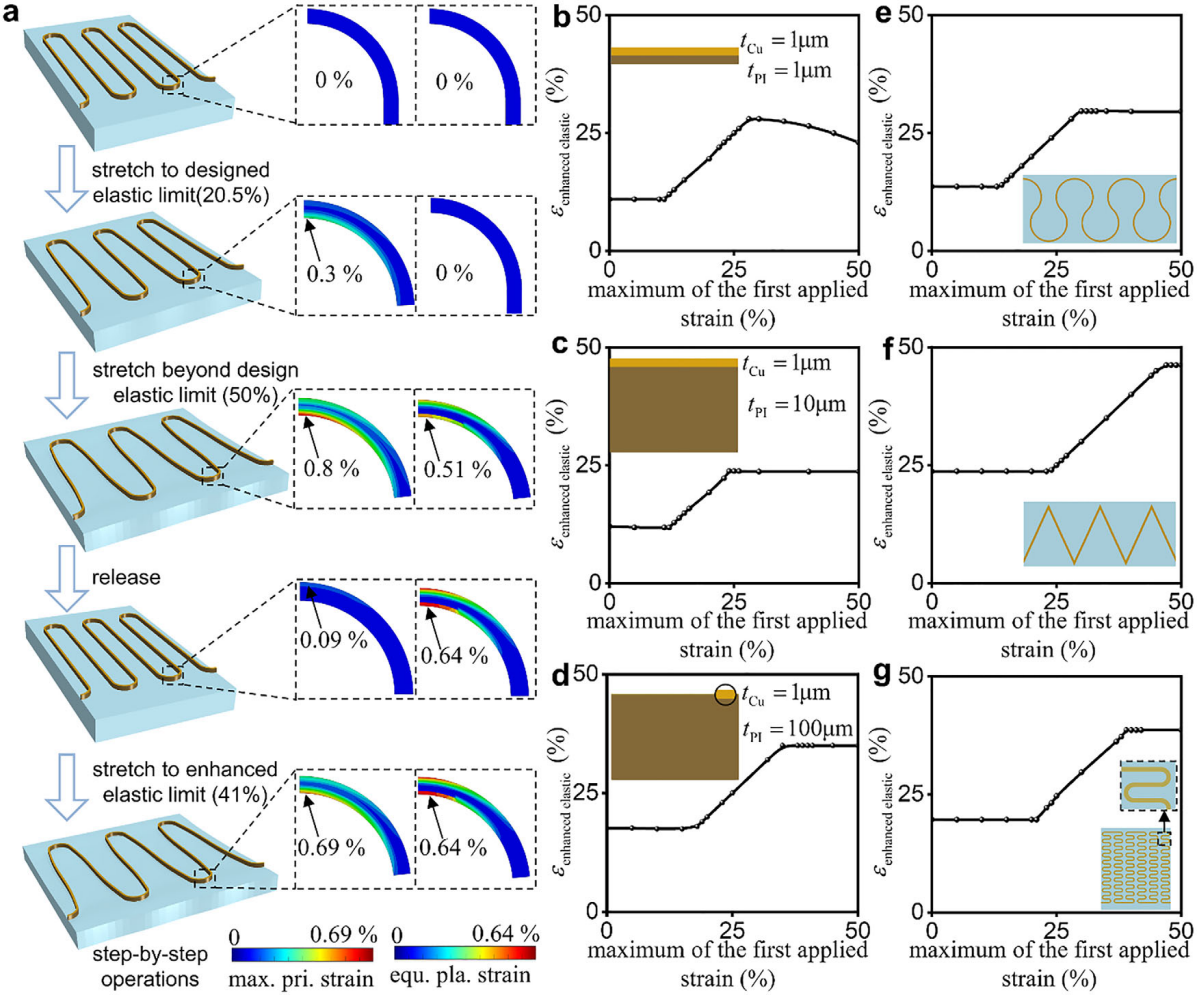
Combination of the Laminating Strategy and Overstretch Strategy: a) Chematic diagram of the combination of overstretch strategy and laminating strategy, maximum principal strain and equivalent plastic strain distribution diagram of key parts. Results of FEA for the combination of overstretch strategy and laminating strategy with PI thickness of b) 1µm, c) 10µm, d) 100µm. Results of the enhanced elastic stretchability of e) horseshoe, f) zigzag, g) fractal interconnects with thick PI.

As a foundational and novel approach, the laminating strategy holds significant potential for combination with the overstretch strategy. Figure 4a shows the operation of the combination of these two strategies. The first column shows the step‐by‐step operation of their combination, the second and third column represent the contours of the maximum principal strain and the equivalent plastic strain of the key parts of the interconnect by FEA. The detailed operations are as follows: 1) The laminated interconnect consisting of Cu/PI with the thickness of 1µm/100µm is transferred and bonded to the substrate. The entire structure is stress/strain‐free. 2) The maximum stress at the cross‐section of the arc vertex reaches the yield stress σ_*s*_, corresponding to the structure reaching the designed elastic stretchability of 20.5%. During this process, the deformation of the interconnect remains pure elasticity without any plasticity. 3) The structure is further stretched to 50%, inducing plastic behavior at the cross‐section of the arc vertex in the Cu layer. The maximum strain and the equivalent plastic strain increase to 0.8% and 0.51% with the increasing of stretching, respectively. Although a local plastic deformation exists in the Cu layer, the PI layer and remaining Cu regions can withstand the additional stretching without damage. 4) Releasing the overstretch, the substrate nearly recovers its initial state, thereby restoring the interconnect back to its original configuration. With the release, the plastic regions of Cu experience elastic unloading, elastic reverse loading and plastic reverse loading sequentially. The maximum principal strain decreases to 0.09%, while the equivalent plastic strain continues to increase to 0.64%. 5) After the complete execution of the operation, the structure can be reloaded, and the elastic stretchability in this stage reaches 41%, representing a double increase over the original design. The result demonstrates that the laminating strategy can be effectively combined with the overstretch strategy, leading to further enhancement for the elastic stretchability.

To investigate the effects of the laminating strategy on overstretch strategy in the combination, the elastic stretchability of the interconnects with varying *t*_PI_ is evaluated through FEA. Figure 4b–d presents the elastic stretchabilities of the interconnects with *t*_PI_ = 1/10/100µm as a function of the first applied strain. The elastic stretchability is not enhanced when the first applied strain does not exceed the designed elastic stretchability ε_overstretch_ ≤ 11.0%/ε_overstretch_ ≤ 11.9%/ε_overstretch_ ≤ 19.7%. If the maximum of the first applied strain is between one and two times the designed elastic stretchability 11.0% < ε_overstretch_ ≤ 22.0%/11.9% < ε_overstretch_ ≤ 23.8%/19.7% < ε_overstretch_ ≤ 39.4%, the elastic stretchability is enhanced to the first applied strain ε_enhanced elastic_ = ε_overstretch_. If the first applied strain exceeds twice the designed elastic stretchability ε_overstretch_ > 22.0%/ε_overstretch_ > 23.8%/ε_overstretch_ > 39.4%, the maximum enhanced elastic stretchability can reach or even exceed twice the designed elastic stretchability, i.e., ε_max enhanced elastic_ = 26.5%/ε_max enhanced elastic_ = 23.8%/ε_max enhanced elastic_ = 39.5%, respectively. Notably, distinct from the stable trend of the interconnects with thick PI, the enhanced elastic stretchability of interconnects with thin PI finally decreases gradually with increasing first applied strain. To analyze the underlying mechanism, the interconnects with thin PI undergo significant out‐of‐plane buckling during overstretch, in contrast to the in‐plane bending of interconnects with thick PI, as shown in Figure S2. Thus, the laminating strategy can affect the enhanced elastic stretchability through modifying the deformation mode of interconnects during overstretch.

To explore the synergetic influence of overstretch strategy and laminating strategy on the elastic stretchability of interconnects with different geometries, FEA is performed for the representative horseshoe/zigzag/fractal structures with *t*_PI_ = 100µm, as shown in Figure S8. As shown in Figure 4e–g, the designed elastic stretchability of the horseshoe/zigzag/fractal structures are 14.8%/23.7%/22.2%, and the corresponding enhanced elastic stretchabilities are 29.6%/47.2%/44.4%, which are twice the corresponding designed elastic stretchabilities. These results show that the overstretch strategy is effective for thick interconnects with various geometrical layouts. Further analysis reveals that, for the horseshoe/zigzag/fractal structures with *t*_PI_ = 1µm, the designed elastic stretchability of 9.8%/11.0%/7.9%and the enhanced elastic stretchability of 16.2%/23.2%/16.4% are both lower than those of interconnects with *t*_PI_ = 100µm (Figure S9a–c). The above FEA results demonstrate that the combination of the overstretch strategy and laminating strategy exhibits synergetic enhancement effects for interconnects with various geometrical layouts, including serpentine, horseshoe, zigzag, and fractal structures.

### 2.3 Application of the Laminating Strategy to Smart Tires with Hard Elastic Substrate

**FIGURE 5:**
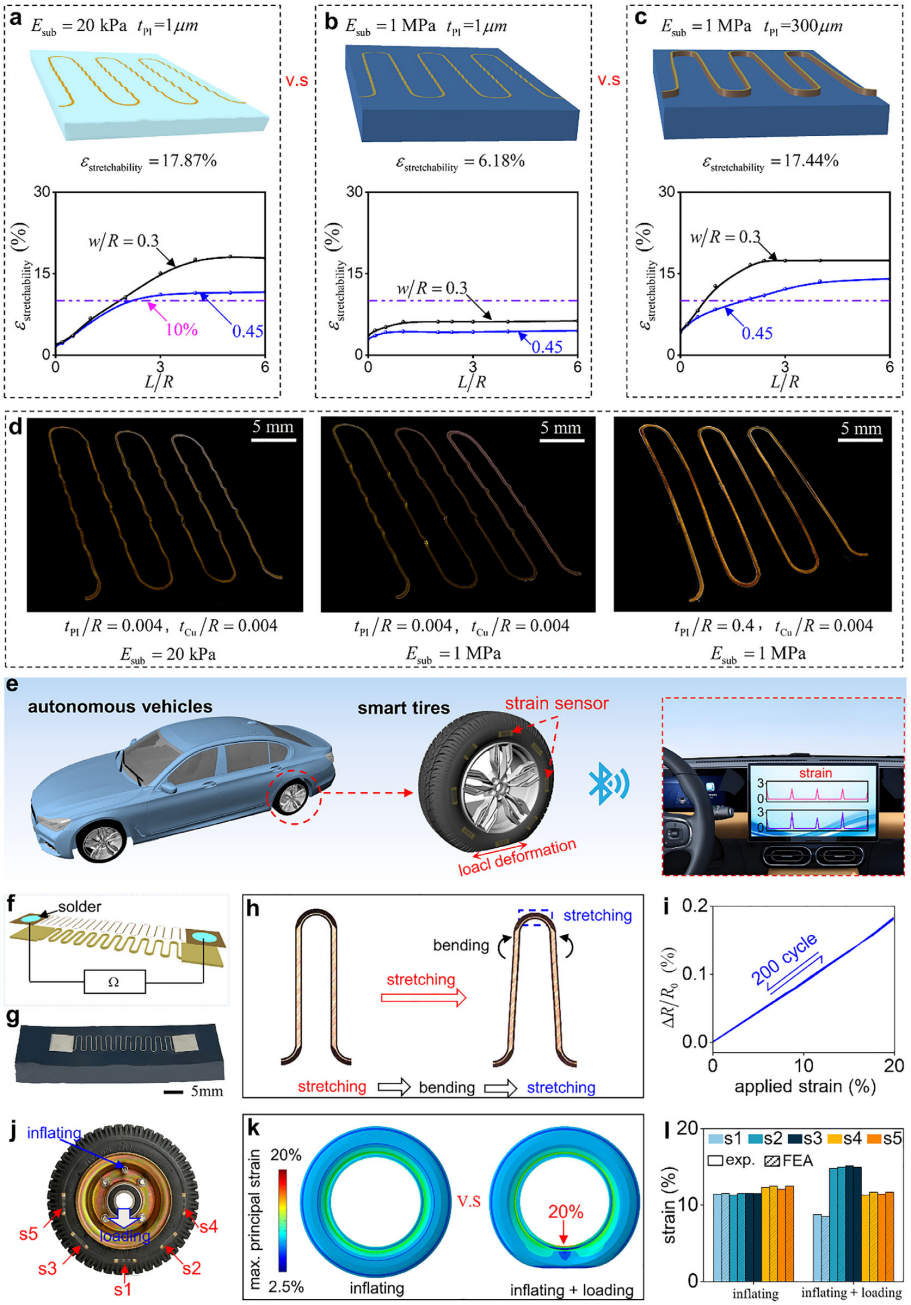
Application of the Laminating Strategy on hard Elastic Substrate: a) Deformation modes of stretchable electronics based on soft elastic substrates and effect of geometric parameters on their elastic stretchability. b) Deformation modes of stretchable electronics based on hard elastic substrates and effect of geometric parameters on their elastic stretchability. c) Deformation modes of thick PI‐based stretchable electronics on hard elastic substrates and effect of geometric parameters on their elastic stretchability. d) Experimental validation of substrate effects on deformation modes in stretchable electronics and optimization of laminating strategy. e) The application of large strain sensors in autonomous driving and smart tires. f) Schematic diagram of the structure of stretchable sensors based on the laminating strategy. h) Schematic diagram of the working principle of stretchable sensors based on the laminating strategy. i) Electrical response of stretchable sensors based on the laminating strategy during cyclic loading‐unloading experiments. j) Schematic diagram of smart tires. h) Strain distribution after tire inflation under no‐load and load conditions. l) Strain measurement results of smart tires.

To accommodate diverse application scenarios, the stretchable structures are usually bonded on the substrates of different materials. However, the elastic stretchability of structures decreases significantly as the elastic modulus of the substrate increases, which severely limits their practical applications. To further examine the phenomenon, FEA is performed to evaluate the influence of geometric parameters on the elastic stretchability of interconnects bonded on substrates with different elastic modulus, as shown in Figure 5a,b. The results indicate that compared to soft elastic substrate with *E*_sub_ = 20kPa, the enhancement effect in elastic stretchability via geometric parameter optimization is constrained for hard elastic substrate with *E*_sub_ = 1MPa, which remains insufficient to accommodate the large‐strain requirement exceeding 10% in practical applications. To address the issue, the influence of laminating strategy on the elastic stretchability of interconnects on the hard elastic substrate is investigated. Figure 5c demonstrates that the laminating strategy enables significant enhancement in the elastic stretchability of interconnects on hard elastic substrate combined with geometric parameter optimization. The observed enhancement originates from the transformation of deformation modes induced by the laminating strategy, which has been further experimentally validated, as shown in Figure 5d. The above analysis demonstrates that the laminating strategy is effective for enhancing the elastic stretchability of interconnects bonded on hard elastic substrates.

As illustrated in Figure 5e, with the rapid proliferation of autonomous vehicles [34, 35], there is an increasing demand for higher safety standards in tires. Although conventional tire pressure monitoring systems effectively mitigate the risk of tire blowouts caused by abnormal tire pressure, their inability to detect local deformation of tire remains a critical deficiency, which may lead to delayed warnings of tire failure caused by excessive large local deformation, thereby posing significant safety hazards. Therefore, it is of great significance to develop smart tires integrated with multiple strain sensors for detecting large local strains. The stretchable strain sensors [15] proposed by our group have been widely adopted in various large‐deformation scenarios due to their advantages of wide measurement range, high repeatability, and excellent linearity. However, the integration of the stretchable strain sensors into smart tires for large deformation detection remains impractical due to the high elastic modulus of tire materials. The application of laminating strategy to the stretchable strain sensor can effectively address this issue. Figure 5f,g presents the schematic illustration and image of the stretchable strain sensor with the laminating strategy, consisting of an off‐axis serpentine Cu layer and a thick PI layer (see Figure S11). The sensing mechanism of the stretchable strain sensor is shown in Figure 5h and Figure S12: Due to the laminating strategy, the sensor primarily undergoes in‐plane bending deformation. The stretching applied strain to the sensor is transferred, through the bending of arc, to a stretching strain of the narrow off‐axis Cu layer, ultimately inducing resistance changes in the sensor. Figure 5i shows relationship between the relative resistance change Δ*R*/*R*_0_ and the applied strain of the stretchable strain sensor bonded on a 35 × 10 × 3mmtire samples during 200 loading‐unloading cycles (see Note S5 and Figure S14), which exhibits extremely high repeatability. To validate the applicability of the sensor in smart tires, five sensors are attached to the tire as shown in the Figure 5j. Subsequently, based on relationship established in Figure 5i, the local strain of the tire is measured by these sensors under inflated and inflated‐loaded conditions. In parallel, FEA is conducted to analyze the strain distribution of the tire under inflated and inflated‐loaded conditions (see Figure 5k) for experimental verification. As shown in Figure 5l, the experimental results show excellent agreement with FEA results. Moreover, the stretchable strain sensors s2 and s3 effectively detect excessive local strain in the tire caused by abnormal loading. Thus, the laminating strategy enables stretchable strain sensors to detect abnormal local deformations in smart tires with high elastic modulus.

## 3 Conclusion

In this study, we propose a laminating strategy to manyfold enhance the elastic stretchability of stretchable electronics. The theoretical, numerical, and experimental results collectively prove that this strategy of laminating a thick organic polymer layer (e.g., 300‐µ*m* ‐thick PI layer) to the thin inorganic stretchable structure (e.g., 1‐µ*m* ‐thick Cu layer) induces a transformation of the deformation mode from out‐of‐plane buckling to in‐plane bending, thereby achieving significant enhancement of the elastic stretchability. FEA results of the effects of geometric parameters on laminating strategy reveal that the structural design with larger dimensionless length and smaller dimensionless width can achieve better enhancement effect. Moreover, the laminating strategy can also be combined with overstretch strategy to further improve the elastic stretchability of stretchable structures with various different geometries. The laminating strategy overcomes the constraints imposed by hard elastic substrates on the elastic stretchability. Owing to such performance, this strategy enables stretchable sensors to measure local large strains in smart tires with high elastic modulus. This strategy provides a fundamental and innovative guidance for the design and fabrication of stretchable electronics based on extensively adopted fabrication technologies.

Notably, this strategy exhibits certain limitations in terms of dimensions and materials: 1) Taking serpentine interconnects as an example, dimensionally, when the straight segment of the interconnect is short, the strategy provides minimal enhancement in elastic stretchability, as shown in Figure 3a. This is because the thick polymer lamination has a limited influence on the deformation mode of interconnects with short straight segments. 2) Material‐wise, as the elastic modulus of the substrate increases, the effectiveness of this strategy in enhancing the elastic stretchability of interconnects decreases. For soft elastic substrates, the strategy increases the elastic stretchability of the interconnect to 38.5% (Figure 2b), whereas for stiff elastic substrates, the improvement is only 17.5% (Figure 5c). Therefore, in practical applications, such dimensional and material conditions should be avoided to ensure optimal performance of this strategy.

In addition, the further optimization of the laminating strategy is discussed: 1) The interfacial delamination is a critical factor influencing the elastic stretchability in the design of stretchable electronics. As shown in Figure S16, the distribution of interfacial shear stress shear stress distribution at the PI/Cu interface and the PI/substrate interface of serpentine interconnects composed of 300‐µm PI and 1‐µm Cu under different tensile strains is calculated by FEA. The results indicate that as the stretching strain increases, the interfacial shear stress correspondingly rises. Once it exceeds a threshold, the stretchable electronic may fail, which further supports the conclusion. This represents a significant and systematic challenge. In future work, based on the existing structural design, we will systematically enhance the elastic stretchability of stretchable electronics by improving interfacial adhesion through methods such as material surface modification, interfacial layer design, and adhesion layer optimization. Moreover, more comprehensive FEA and experiments are expected to reveal the underlying failure mechanisms of the interfacial delamination. 2) While we have preliminarily identified the factors influencing the deformation modes (including mechanical parameters of the interconnect and material properties of the substrate), a quantitative analytical model that precisely describes the relationship between these factors and the deformation modes has not yet been established. Developing such a model remains an important direction for our future work, as it would further enhance the predictability and generalizability of the proposed strategy. 3) In practical applications, although we applied this strategy in strain sensors and fabricated a simple smart tire prototype to verify its feasibility on hard elastic substrates, both experimental and FEA results indicate that the distribution of strain sensors is crucial for accurately capturing the state information of the tire. Therefore, in future work, we will conduct more research on the distribution of strain sensors to optimize their layout, aiming to develop smart tires capable of acquiring richer state information under real operating conditions.

## 4 Experimental Section

### 4.1 Preparation of Serpentine Interconnects Bonded on the Substrate

Note that, for ease of observation, the geometric dimensions of the serpentine interconnects have been scaled up by a factor of 10 in equal proportion. The detailed fabrication process is as follows: 1) Preparation of composite materials. Cu/PI composite materials are prepared by the High Power Impulse Magnetron Sputtering (HiPIMS) technique with a copper plate (99.99%) as target and 10/3000‐µm‐thick PI as substrate. 2) Preparation of serpentine interconnects. The serpentine interconnects are fabricated by cutting the above composites using a P‐second laser precision machining system (DCT‐DL 566 PU, China, as shown in Figure S12). The laser power is adjusted to 75% of the maximum power, and 300 cycles of laser cutting are performed. 3) Transfer of serpentine interconnects. The fabricated serpentine interconnects are transferred onto an elastic substrate using water‐soluble tape. After the adhesive is cured, the water‐soluble tape is immersed in water to dissolve and remove it. The interconnect bonded on the substrate is completed.

### 4.2 FEA of the Laminating Strategy

The commercial software Abaqus is used for FEA to validate the laminating strategy. The elastic substrate is considered as a hyperelastic material, described by the Mooney‐Rivlin model, and the detailed parameters refer to Table S1. PI is regarded as a linear elastic material with a Young's modulus of 2.5 GPa and a Poisson's ratio of 0.34. Cu is considered to be a linear elastic‐plastic material with a Young's modulus of 124 GPa, a Poisson's ratio of 0.33, and a yield stress of 372 MPa, corresponding to an elastic strain range of 0.3%. The substrate adopts C3D8 unit, PI and Cu adopt C3D8R unit.

### 4.3 Fabrication Process of the Stretchable Strain Sensor

As shown in Figure S13, the fabrication processes of the stretchable strain sensor are as follows: 1). Cu/PI composite materials are prepared by the HiPIMS technique with a copper plate (99.99%) as target and 300‐µm‐thick PI as substrate. 2). The Cu layer is patterned to the off‐axis serpentine structure by a standard photolithography process (including coating photoresist, prebaking, UV exposure, development, corrosion of ferric chloride solution, and removal of photoresist), as shown in Figure S11. 3). The sandwich structure is patterned to get the stretchable strain sensor by a UV picosecond laser (DL566PU, DCT, China). 4). Two copper wires are soldered to both ends of the serpentine structure as the electrodes to connect with the outside circuit.

### 4.4 Experiment to Measure Strain of Tire under Inflated and Inflated‐Loaded Conditions by Stretchable Strain Sensor

The stretchable strain sensor is attached to the outer side of the tire, as shown in Figure 5j. The tire is inflated using an air pump until the internal pressure reaches 250 kPa, while the signal acquisition system (4294A, Keysight, USA) is used to measure the changes in the sensor's resistance. Subsequently, a load is applied at the center of the tire, causing it to deform by 20 mm, and the signal acquisition system is again used to measure the changes in the sensor's resistance.

## Funding

The National Natural Science Foundation of China (grant 12172359); the Open Research Project of Zhongke Technology Achievement Transfer and Transformation Center of Henan Province (2025113).

## Conflicts of Interest

The authors declare no conflicts of interest.

## Supporting information




**Supporting File**: advs74030‐sup‐0001‐SuppMat.docx.

## Data Availability

Research data are not shared.
