# Supplementary material for: A Laminating Strategy to Manyfold Enhance the Elastic Stretchability of Stretchable Electronics
Source: Adv Sci (Weinh). 2026 Jan 25;13(19):e21763. doi: 10.1002/advs.202521763 (PMC13045469; doi:10.1002/advs.202521763)
Supplement: Supplementary file 1 — Supporting File: advs74030‐sup‐0001‐SuppMat.docx. [file ADVS-13-e21763-s001.docx]

Supporting Information

**A Laminating Strategy to Manyfold Enhance the Elastic Stretchability of Stretchable Electronics**

*Zanxin Zhou, Xiaolei Wu, Xinkai Xu, Fanming Wang, Shuang Li, Huiling Li, and Yewang Su**

Z. Zhou, Prof. X. Wu, X. Xu, F. Wang, S. Li, Prof. H. Li, Prof. Y. Su

State Key Laboratory of Nonlinear Mechanics, Institute of Mechanics, Chinese Academy of Sciences, Beijing 100190, China

E-mail: yewangsu@imech.ac.cn

Z. Zhou, Prof. X. Wu, X. Xu, F. Wang, Prof. H. Li, Prof. Y. Su

School of Engineering Science, University of Chinese Academy of Sciences, Beijing 100049, China

X. Xu

Department of Bioengineering, University of California, Los Angeles, Los Angeles, CA 90095, USA

S. Li

Institute of Biomechanics and Medical Engineering, Applied Mechanics Laboratory, Department of Engineering Mechanics, Tsinghua University, Beijing 100084, China

Prof. Y. Su

Zhongke Technology Achievement Transfer and Transformation Center of Henan Province, Changyuan County, Henan 453000, China

**This PDF file includes:**

Supplementary Text

Note S1-S5

Tables. S1-S2

Figs. S1 to S18

**Supplementary Text**

**Note S1. Calculation of effective bending stiffness $\overline{EI}_{out-of-plane}$ and $\overline{EI}_{in-plane}$.**

**Calculation of effective bending stiffness $\overline{EI}_{out-of-plane}$.**

The effective bending stiffness $\overline{EI}_{out-of-plane}$ governs the out-of-plane deformation of the interconnect, including $\overline{EI}_{x}$ in the straight segments and $\overline{EI}_{r}$ in the arc segments. For a multi-layer film with $t_{i}$ and $E_{i}$ as the thickness and Young's modulus for *i*-th layer, where *i* = 1~n represents the number of each layer from bottom to top, the effective bending stiffness $\overline{EI}_{out-of-plane}$ is given by:

$$\overline{EI}_{out-of-plane}=\overline{EI}_{x}=\overline{EI}_{r}=w\sum_{i=1}^{n} E_{i}t_{i}\left[\frac{t_{i}^{2}}{3}+\left(\sum_{j=1}^{i} t_{j}-y_{neutral}\right)\left(\sum_{j=1}^{i} t_{j}-y_{neutral}-t_{i}\right)\right]$$

where $y_{neautral}=\left[\sum_{i=1}^{n} E_{i}t_{i}\left(2\sum_{j=1}^{i} t_{j}-t_{i}\right)\right]/\left(2\sum_{i=1}^{n} E_{i}t_{i}\right)$ is the effective thickness, *w* represents the width of the interconnect. For the two-layer film consisting of PI and metal layers as shown in figure S3b, the effective bending stiffness becomes the equation in equation .

$$\overline{EI}_{out-of-plane}=\frac{\left(E_{PI}t_{PI}^{2}-E_{m}t_{m}^{2}\right)+4E_{PI}t_{PI}E_{m}t_{m}(t_{PI}+t_{m})^{2}}{12\left(E_{PI}t_{PI}+E_{m}t_{m}\right)}w$$

For most of the metal $E_{m}>>E_{PI}$, this equation can be approximated with

$$\overline{EI}_{out-of-plane}\approx\frac{E_{m}t_{m}^{3}+4E_{PI}t_{PI}(t_{PI}+t_{m})^{2}}{12}w$$

**Calculation of effective bending stiffness $\overline{EI}_{in-plane}$.**

The effective bending stiffness $\overline{EI}_{in-plane}$ governs the in-plane deformation of the interconnect, including $\overline{EI}_{z}$ in the straight segments and the arc segments. For a multi-layer film with $t_{i}$ and $E_{i}$ as the thickness and Young's modulus for *i*-th layer, where *i* = 1~n represents the number of each layer from bottom to top, the effective bending stiffness $\overline{EI}_{in-plane}$ is given by:

$$\overline{EI}_{in-plane}=\overline{EI}_{z}=\frac{w^{3}}{12}\sum_{i=1}^{n} E_{i}t_{i}$$

For the two-layer film consisting of PI and metal layers as shown in figure S3b, the effective bending stiffness becomes the expression in equation .

$$\overline{EI}_{in-plane}=\frac{w^{3}}{12}\left(E_{PI}t_{PI}+E_{m}t_{m}\right)$$

**Note S2. Theoretical analysis for the limit of the elastic stretchability of the serpentine interconnect based on laminating strategy.**

The serpentine interconnect is modeled as the curved Euler-Bernoulli beam. For the straight segment and arc segment, the effective bending stiffness about the *z* axis can be calculated as

$$\overline{EI}_{z,str}=\overline{EI}_{z,\text{arc}}=\frac{w^{3}}{12}\left(E_{PI}t_{PI}+E_{Cu}t_{Cu}\right)$$

where *w* is the width of the serpentine interconnect, $t_{PI}$ and $t_{Cu}$ are the thicknesses of PI and Cu, $E_{PI}$ and $E_{Cu}$ are the Young’s moduli of PI and Cu, respectively. Based on the model in figure S2c, the bending moments about the *z* axis due to the applied force P subjected to the bottom end A is

$$\left\{\begin{matrix} M_{z,arc}=P\left(\frac{L}{2}+R\cos\frac{s}{R}\right)\text{ }for\text{ }0\leq s\leq\frac{\pi R}{2} \\ M_{z,str}=P\left(\frac{L+\pi R}{2}-s\right)\text{ }for\text{ }\frac{\pi R}{2}\leq s\leq\frac{\pi R+L}{2} \end{matrix}\right.$$

where $s\in\left[0,\left(\pi R+L\right)/2\right]$ is the curvilinear coordinate. The curvature increments about the z axis of the serpentine interconnection are subsequently resulted as

$$\left\{\begin{matrix} \Delta\kappa_{z,arc}=\frac{M_{z,arc}}{\overline{EI}_{z,arc}}\text{ }for\text{ }0\leq s\leq\frac{\pi R}{2} \\ \Delta\kappa_{z,str}=\frac{M_{z,str}}{\overline{EI}_{z,str}}\text{ }for\text{ }\frac{\pi R}{2}\leq s\leq\frac{\pi R+L}{2} \end{matrix}\right.$$

where the effective bending stiffness $\overline{EI}_{z,str}$ and $\overline{EI}_{z,arc}$ are given in Equation , for the straight and arc segments, respectively. Integration of the curvature increments in Equation yields the horizontal displacement of the bottom end A

$$u_{A}=\int_{0}^{\pi R/2} \left(\int_{0}^{s} \Delta\kappa_{z,arc}ds\right)\sin\left(\frac{s}{R}\right)ds+\int_{\pi R/2}^{\left(\pi R+L\right)/2} \left(\int_{0}^{\pi R/2} \Delta\kappa_{z,arc}ds+\int_{\pi R/2}^{s} \Delta\kappa_{z,str}ds\right)ds$$

which is related with the applied strain by

$$\varepsilon_{applied}=\frac{u_{A}}{R}$$

On the other hand, the maximum strain $\varepsilon_{\max}$ of the serpentine interconnection occurs at the left end C, which can be obtained as

$$\varepsilon_{\max}=\Delta\kappa_{z,arc}|_{s=0}\frac{w}{2}$$

The combination of the above equations gives the theoretical analysis of the relationship between the applied strain $\varepsilon_{\text{applied}}$ of the serpentine interconnection and the maximum strain $\varepsilon_{\max}$ in the metal layer, by eliminating the applied force P:

$$\varepsilon_{\text{applied}}=2\overline{R}\left(\frac{\overline{L}^{3}}{12\overline{L}+24}+\frac{\pi\overline{L}^{2}+8\overline{L}+2\pi}{4\overline{L}+8}\right)\varepsilon_{\max}$$

When the maximum strain $\varepsilon_{\max}$ of in the metal layer reaches the elastic strain limit of metal $\varepsilon_{\max}=\varepsilon_{\text{limit of metal}}$, the limit of the elastic stretchability $\varepsilon_{\text{limit of interconnect}}$ is obtained by taking into the equation :

$$\varepsilon_{\text{limit of interconnect}}=2\overline{R}\left(\frac{\overline{L}^{3}}{12\overline{L}+24}+\frac{\pi\overline{L}^{2}+8\overline{L}+2\pi}{4\overline{L}+8}\right)\varepsilon_{\text{limit of metal}}$$

**Note S3. FEA of the stretchable strain sensor.**

The commercial software Abaqus is used to study the underlying mechanism of the stretchable strain sensor. The geometric dimensions of the model are illustrated in figure S11. PI is regarded as a linear elastic material with a Young's modulus of 2.5 GPa and a Poisson's ratio of 0.34. Cu is considered to be a linear elastic-plastic material with a Young 's modulus of 124 GPa, a Poisson 's ratio of 0.33, and a yield stress of 372 MPa, corresponding to an elastic strain range of 0.3%. PI and Cu adopt C3D8R unit.

**Note S4. FEA of Tire under Inflated and Inflated-Loaded Conditions.**

The commercial software Abaqus is used to study strain distribution of the tire under inflated and inflated-loaded conditions. The tire is considered as a hyperelastic material, described by the Mooney-Rivlin model, and the detailed parameters refer to table S1. Pressure is applied to the inner surface of the tire to simulate the inflation process. The tire hub is coupled to the center point of the tire, and a downward displacement is applied at the center point to simulate the loading process. The substrate adopts C3D8 unit.

**Note S5. Experiment to measure strain of standard tire specimen by stretchable strain sensor.**

A standard $35\times10\times3\text{ mm}$ tire sample is cut from the tire, and a stretchable sensor is attached to the sample, as shown in figure 5g. The 200 tension-releasing cycles (20% applied strain and $2\text{mm}/\text{s}$ velocity) of the sample is carried out by a programmable tensile testingmachine (ZQ-990A, ZHIQU, China). The changes of the sensor’s resistance during the experiment are measured by the signal acquisition system (4294A, Keysight, USA).

**Table S1. Some parameters of the elastic substrate**

|  | C10 | C01 | D1 |
| --- | --- | --- | --- |
| soft elastic substrate | 0.008053691 | 0.002013423 | 2 |
| hard elastic substrate | 0.134228188 | 0.033557047 | 0.12 |
| tire | 0.402684564 | 0.100671141 | 0.04 |

**Table S2. Some parameters of the polymer.**

|  | elastic modulus | Poisson's ratio |
| --- | --- | --- |
| PTFE | 0.5 | 0.4 |
| PP | 1.5 | 0.35 |
| PI | 2.5 | 0.34 |
| CE | 3 | 0.3 |
| PET | 3.5 | 0.34 |


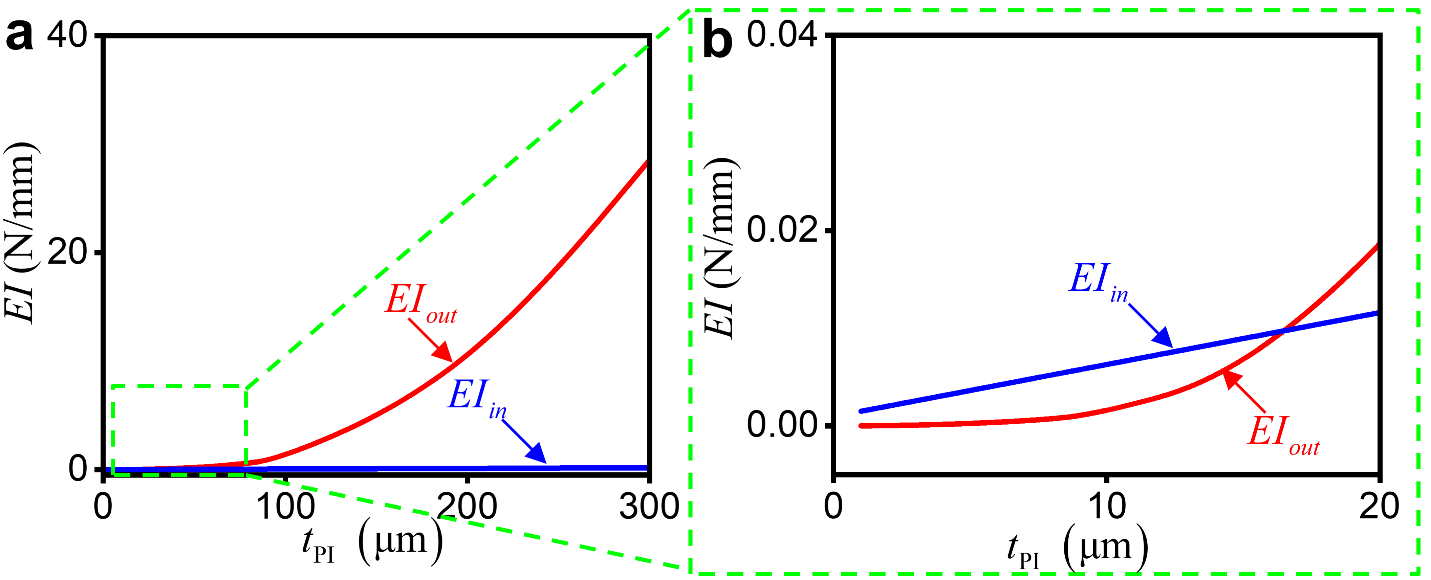


Fig. S1. a) The effect of PI thickness on the equivalent bending stiffness $EI_{out}$, $EI_{in}$ and b) its inset displays.


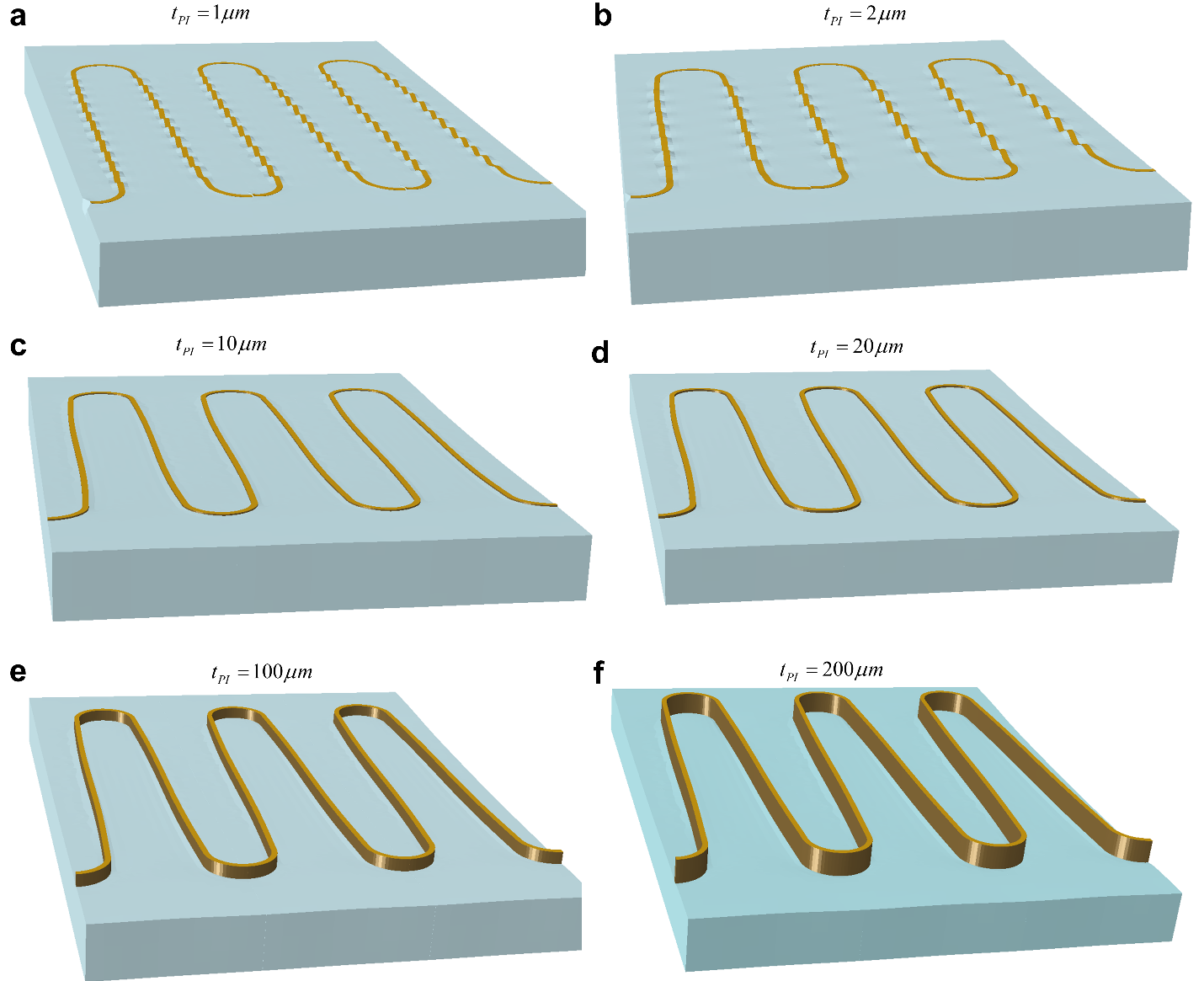


Fig. S2. The effect of different PI thicknesses a)$t_{PI}=1\mu m$, b)$t_{PI}=2\mu m$, c)$t_{PI}=10\mu m$, d) $t_{PI}=20\mu m$, e)$t_{PI}=100\mu m$, f)$t_{PI}=200\mu m$ on the deformation modes of stretchable electronics.

**
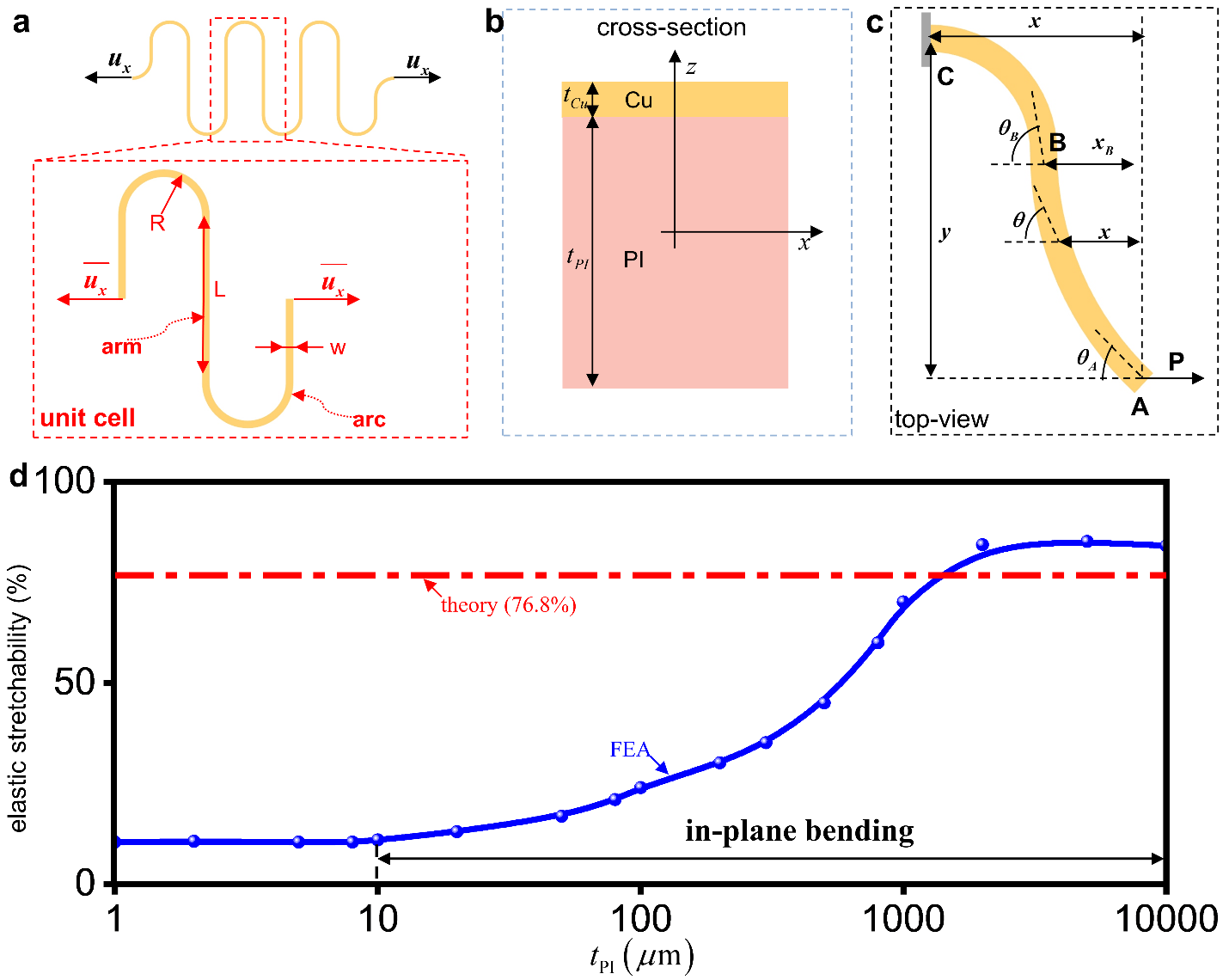
**

Fig. S3. Theoretical analysis of the elastic stretchability of serpentine interconnects prepared via the lamination strategy during the in-plane bending phase. a) Schematic diagram and cross-section of the serpentine interconnect; b) Theoretical analysis model of the serpentine interconnect; c) Comparison between theoretical analysis and finite element simulation results.


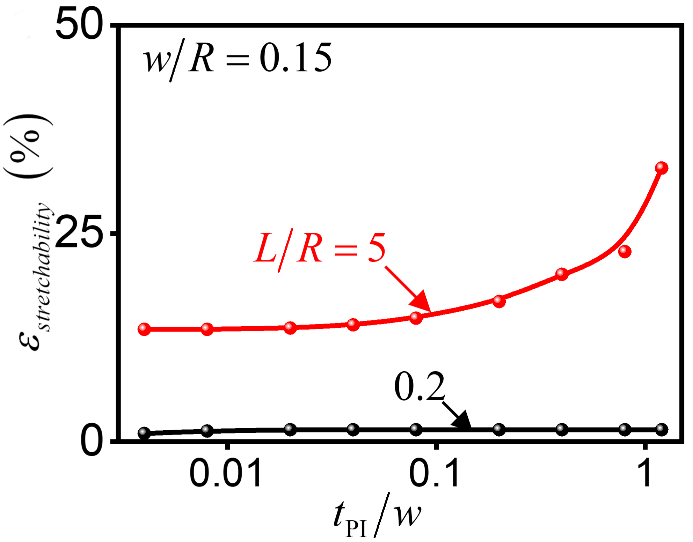


Fig. S4. Elastic stretchability versus $t_{PI}/w$ of interconnects having $L/R=5$ and $L/R=0.2$.


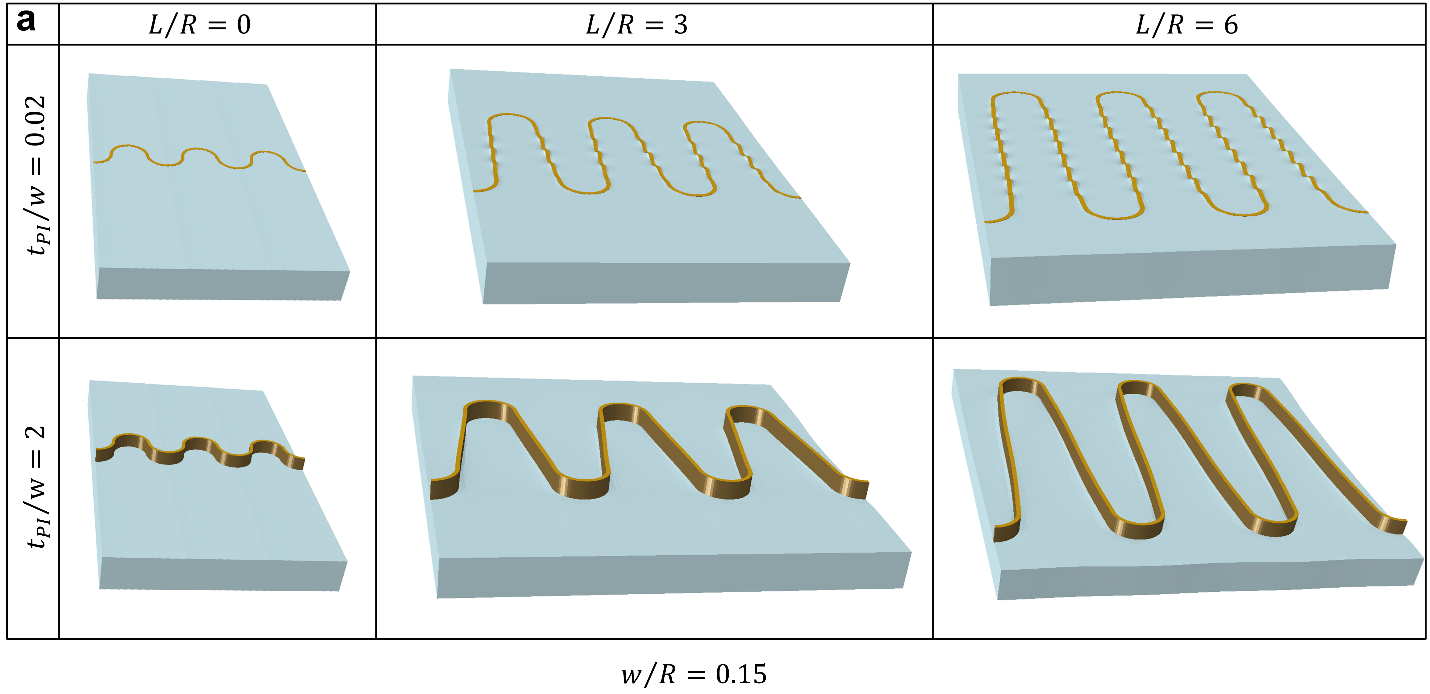


Fig. S5. Observation of different deformation modes for the interconnects having different $L/R$ and $t_{PI}/R$.

**
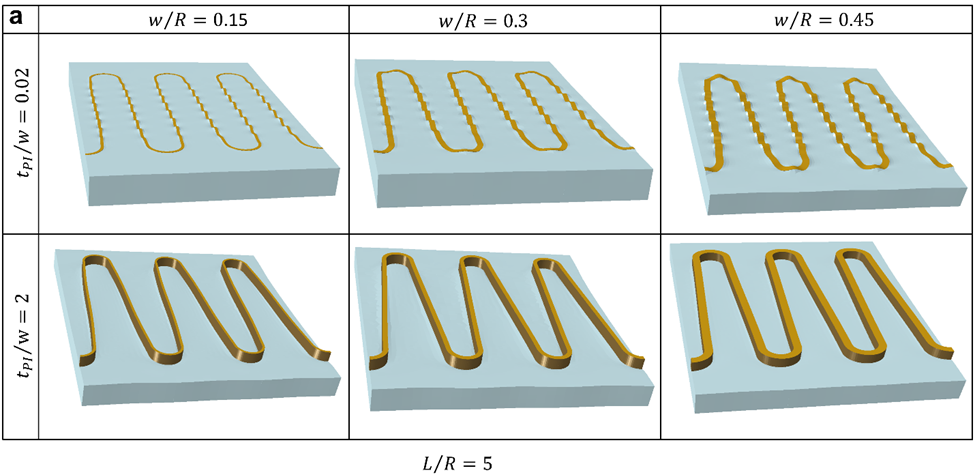
**

Fig. S6. Observation of different deformation modes for the interconnects having different

$w/R$ and ${t_{PI}}/R$.


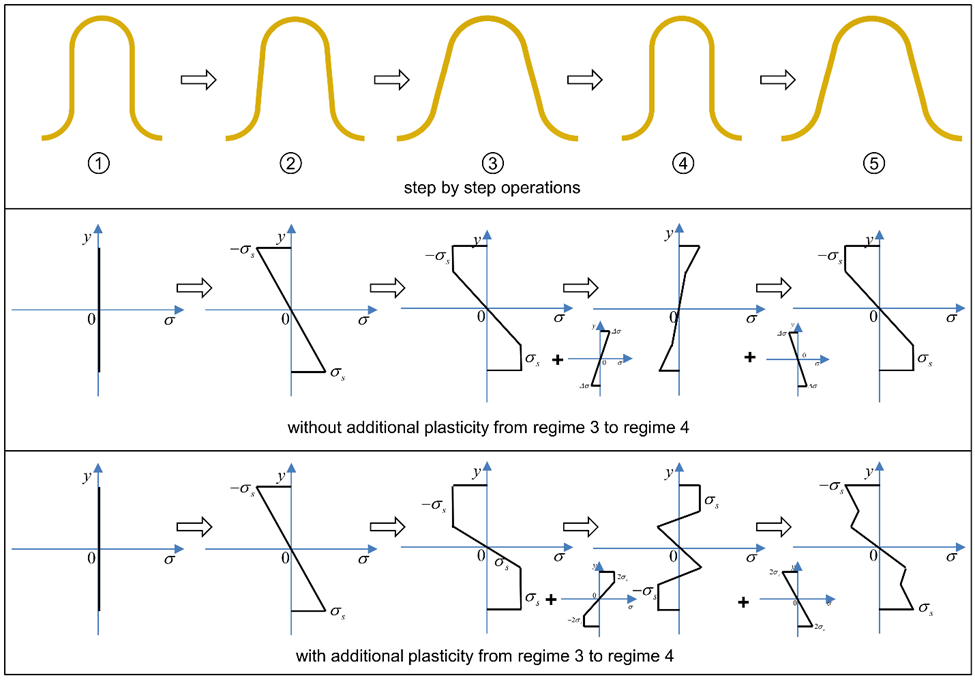


Fig. S7. Schematic illustration of the underlying mechanism of overloading strategy.


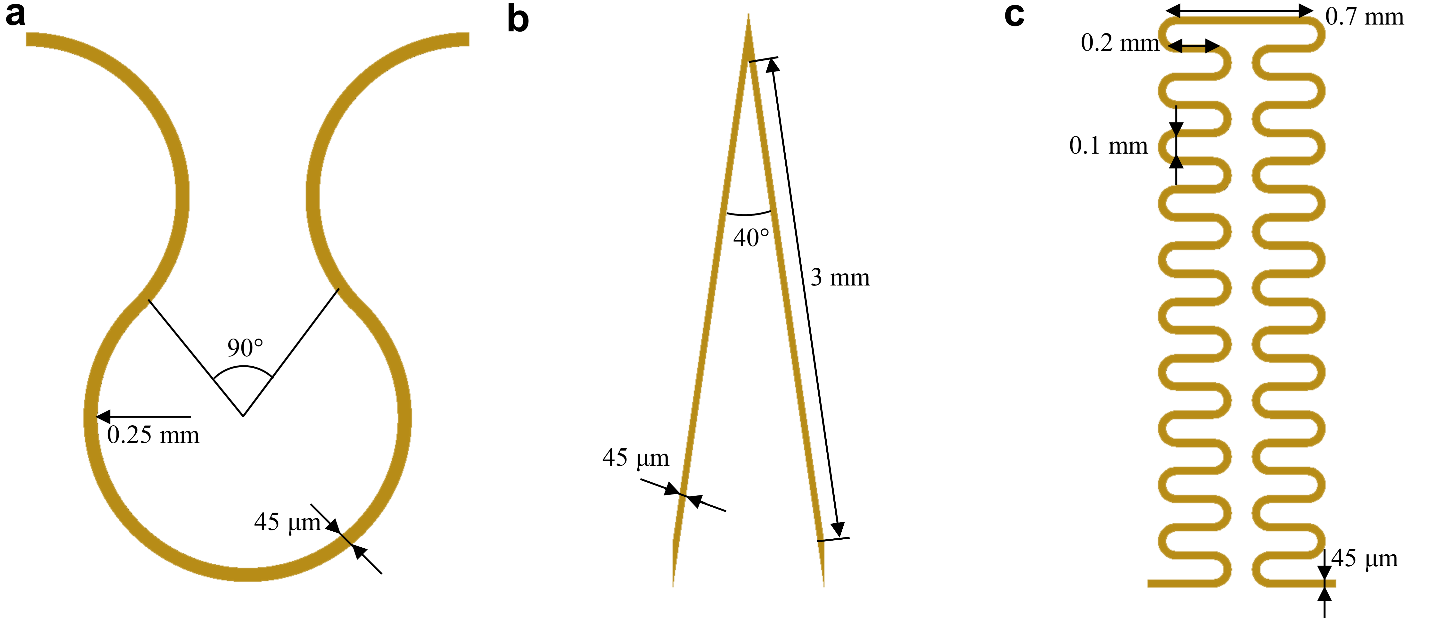


Fig. S8. Geometric structure of a) horseshoe, b) zigzag and c) fractal structures.


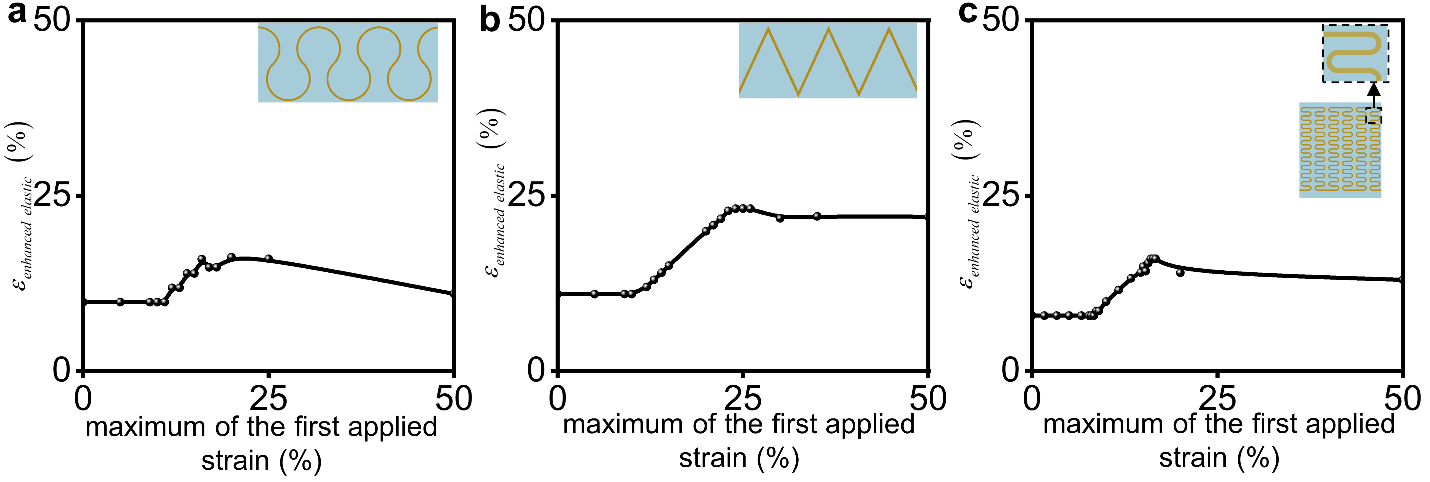


Fig. S9. Results of the enhanced elastic stretchability of a) horseshoe, b) zigzag, c) fractal interconnects with thick PI.


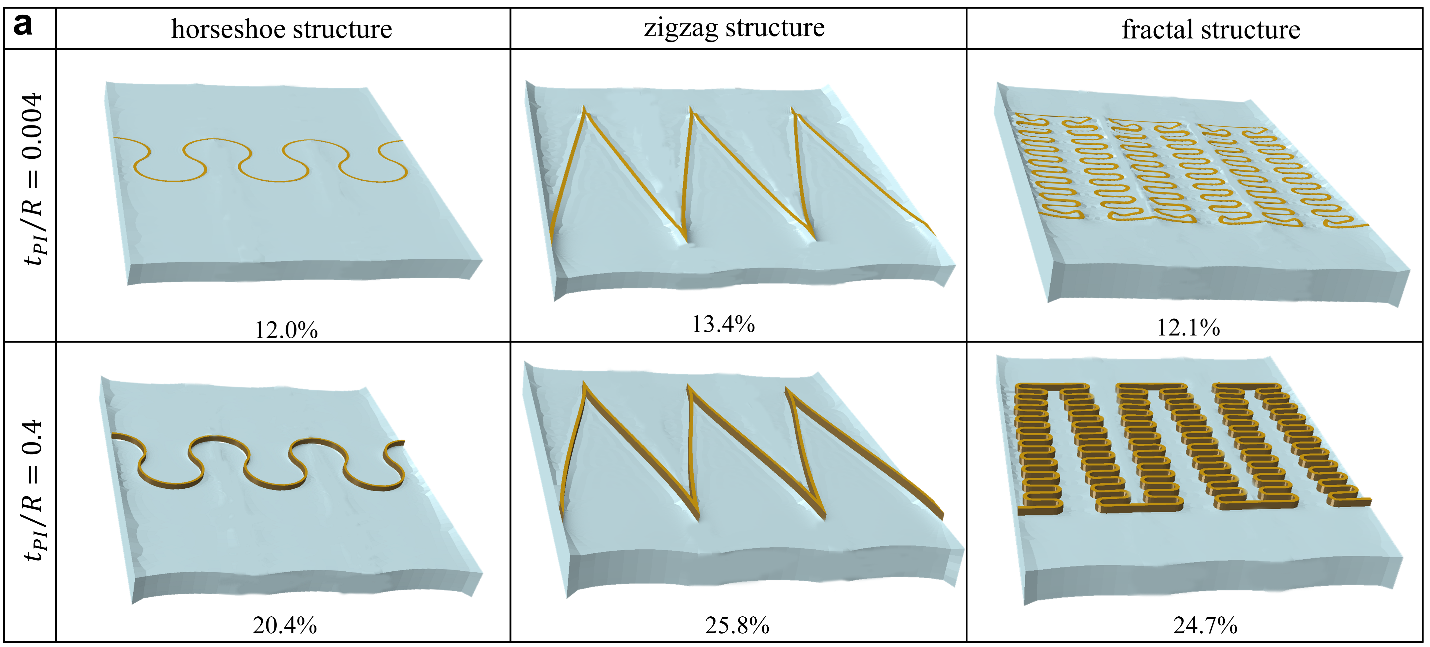


Fig. S10. Observation of different deformation modes for the horseshoe, zigzag and fractal structures.

**
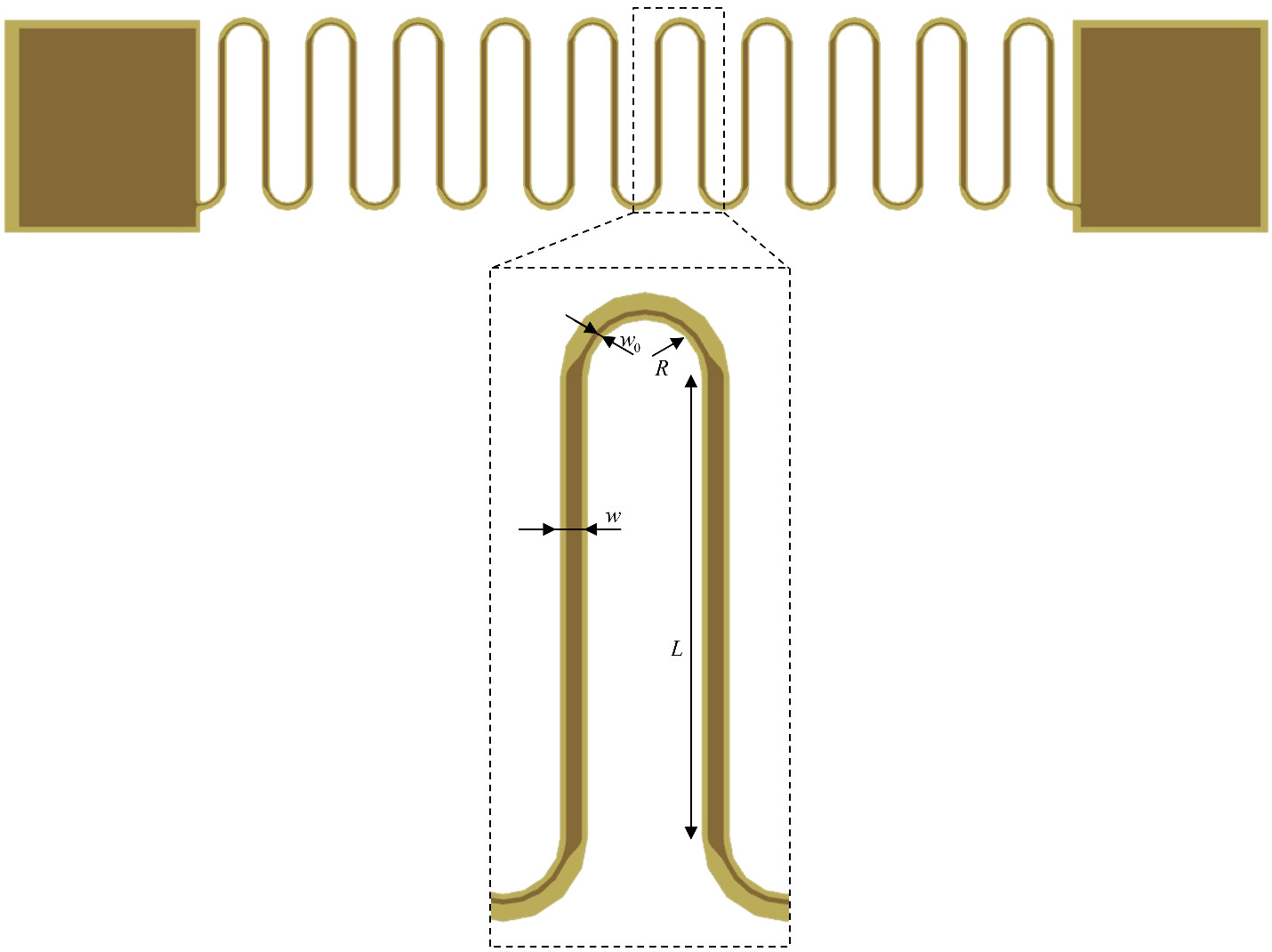
**

Fig. S11. Geometric structure of the sensing mechanism of Stretchable Strain Sensors.

**
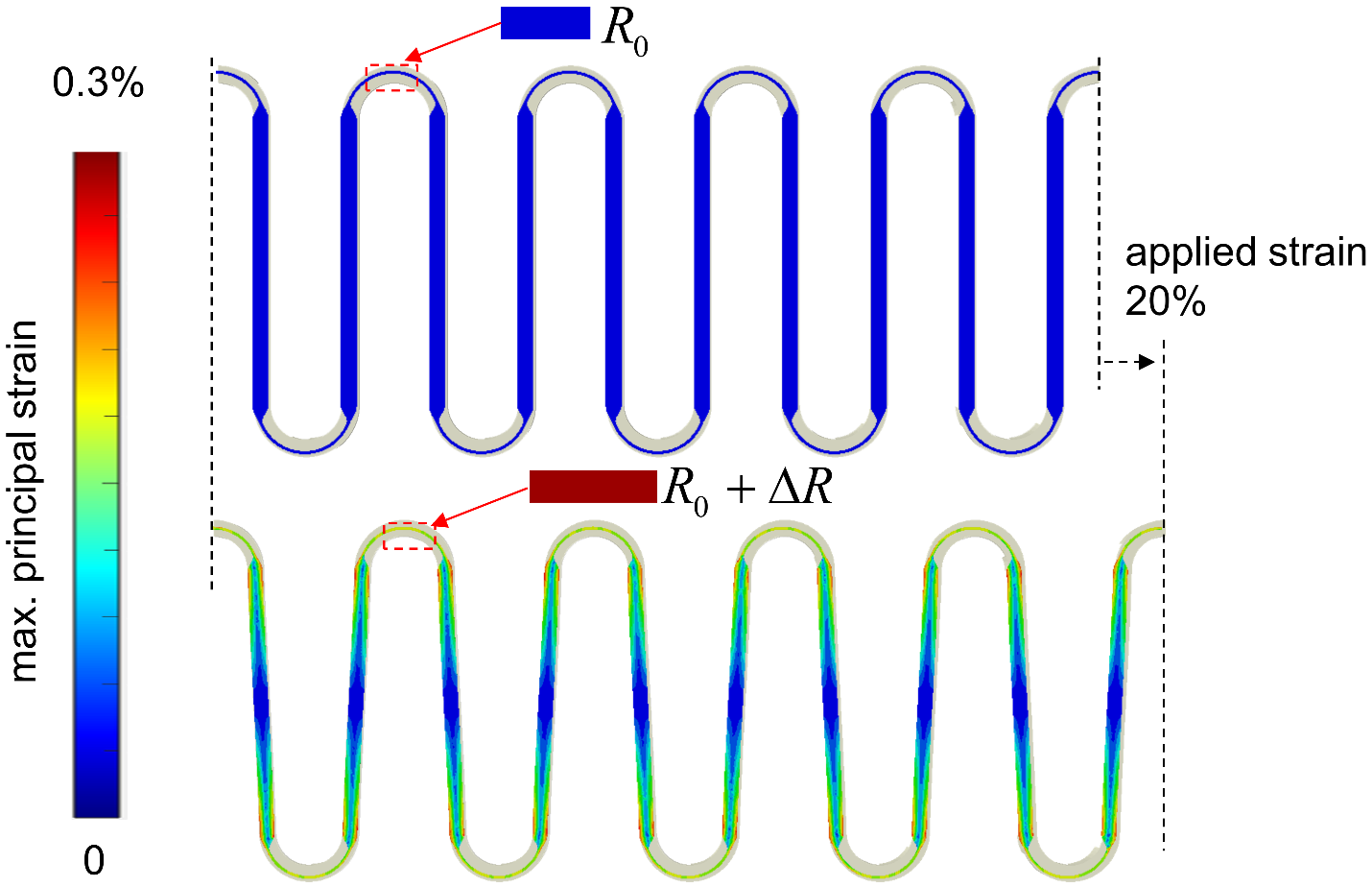
**

Fig. S12. Schematic illustration of the sensing mechanism of Stretchable Strain Sensors.

**
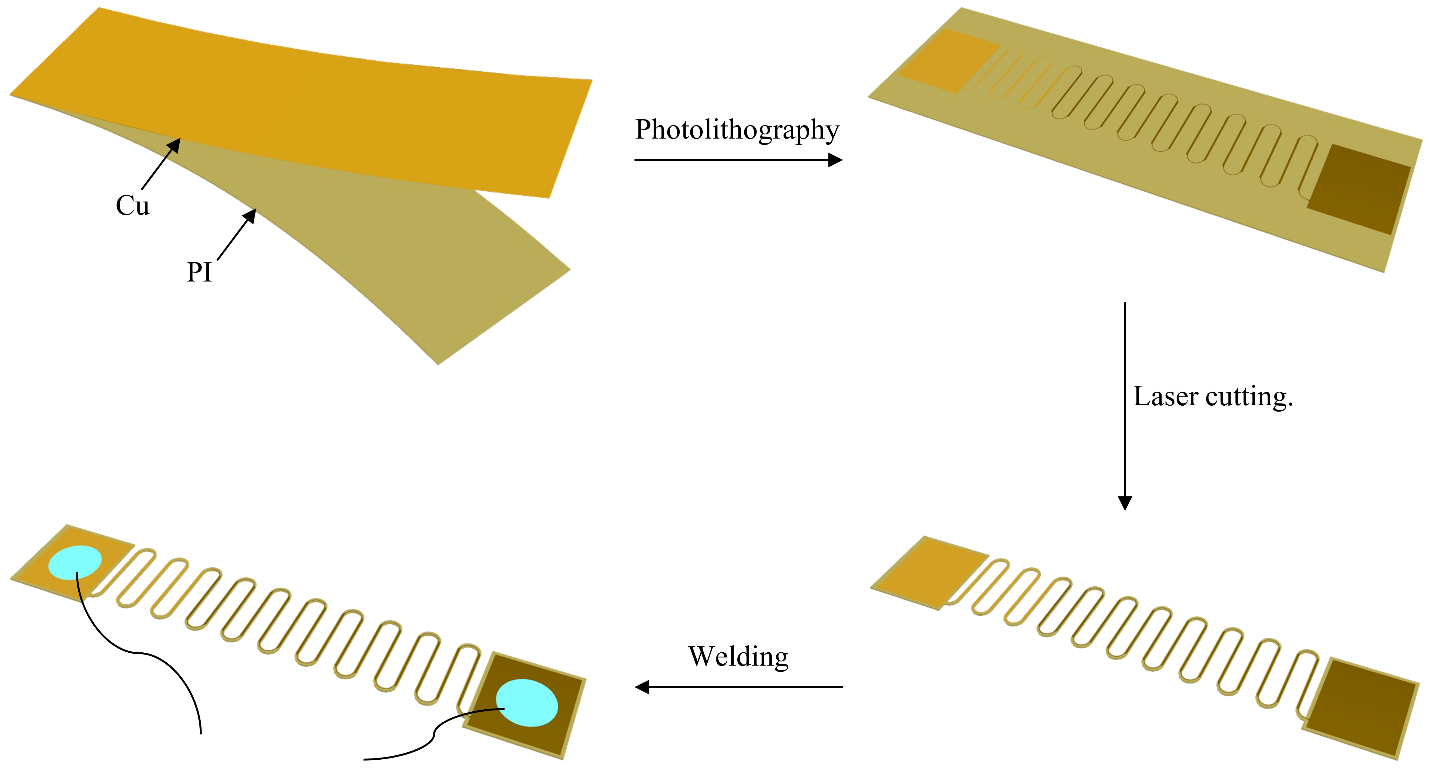
**

Fig. S13. The fabrication process of the stretchable strain sensor.

**
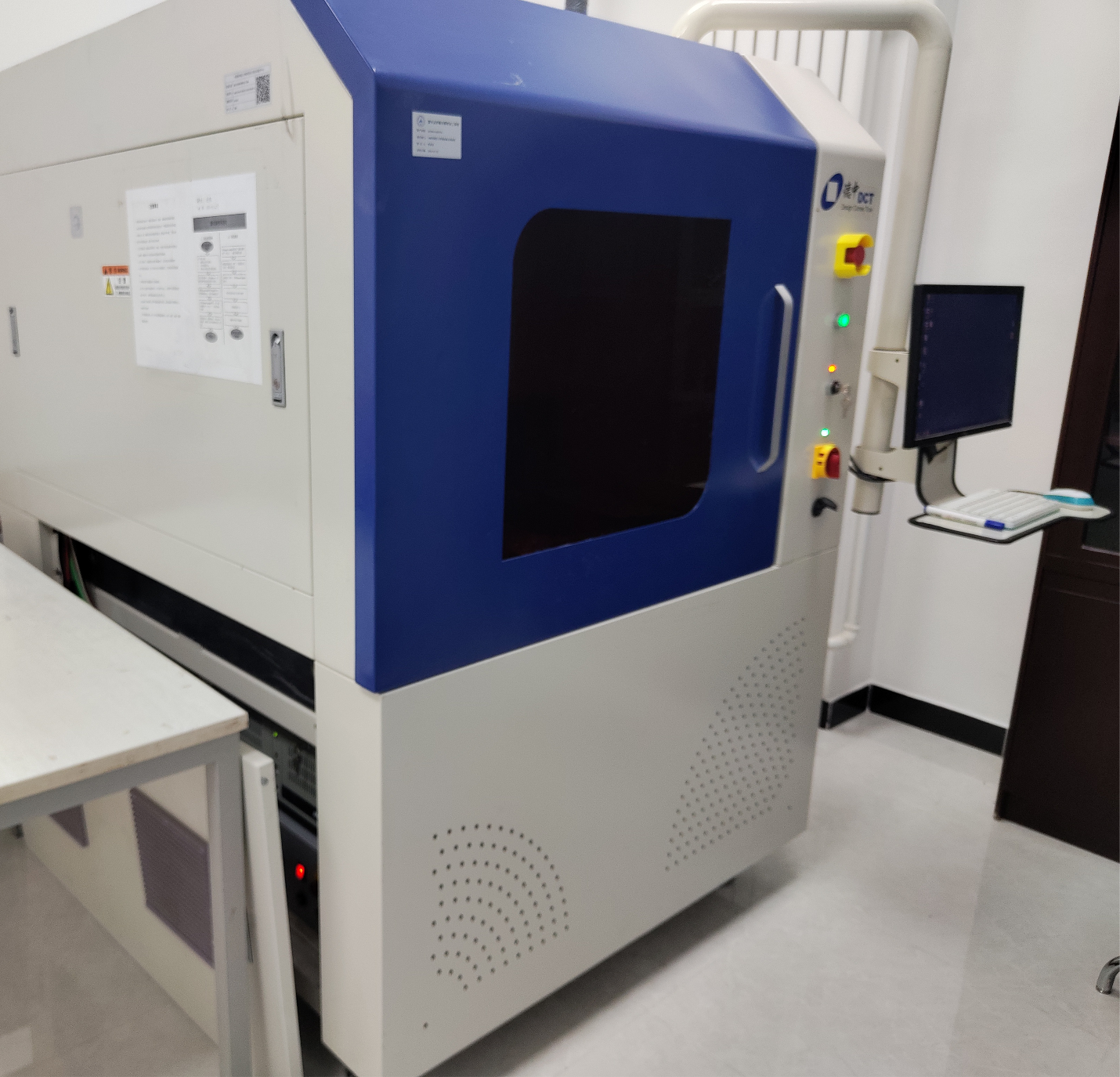
**

Fig. S14. Optical image of P-second laser precision machining system.


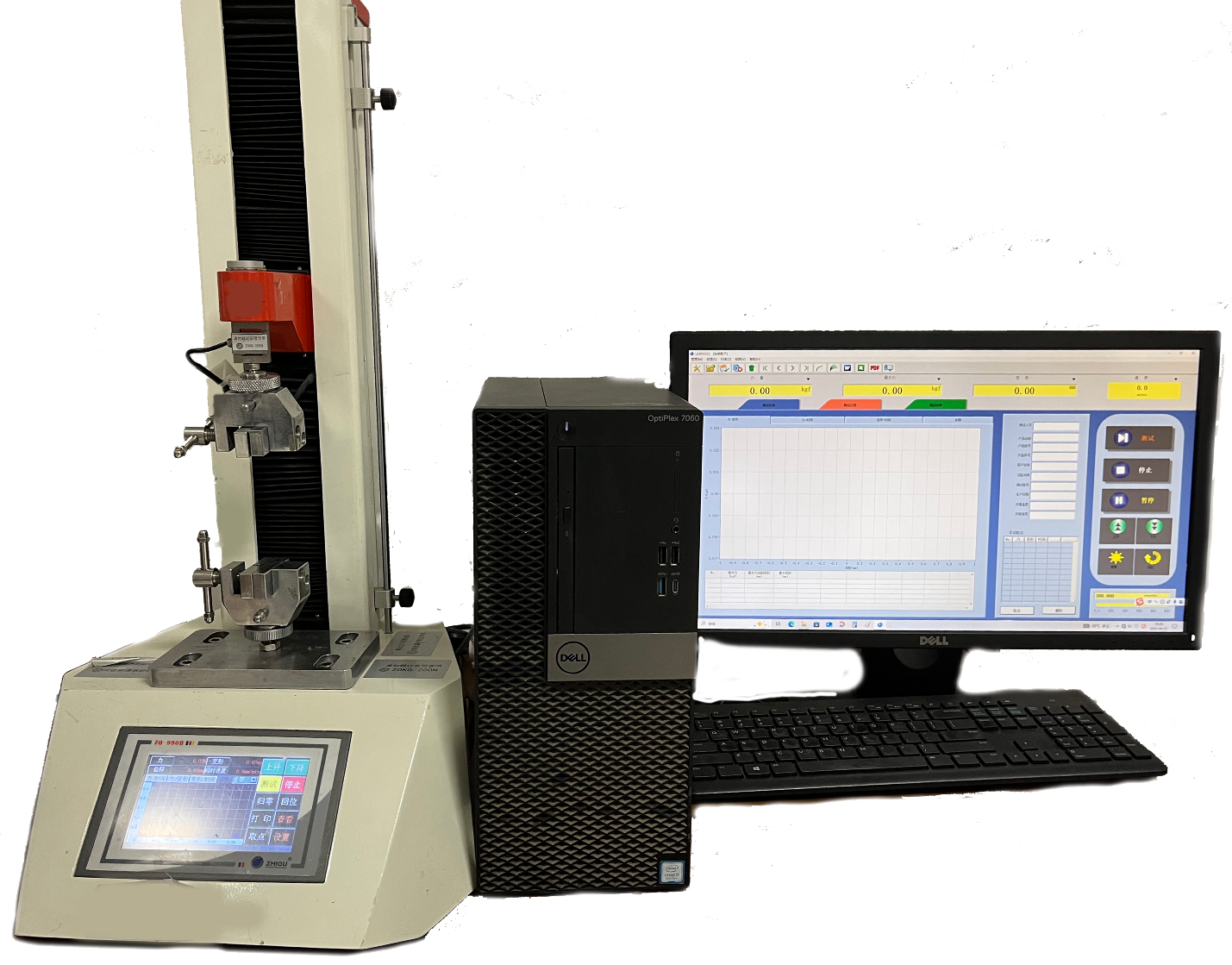


Fig. S15. Optical image of programmable tensile testingmachine.


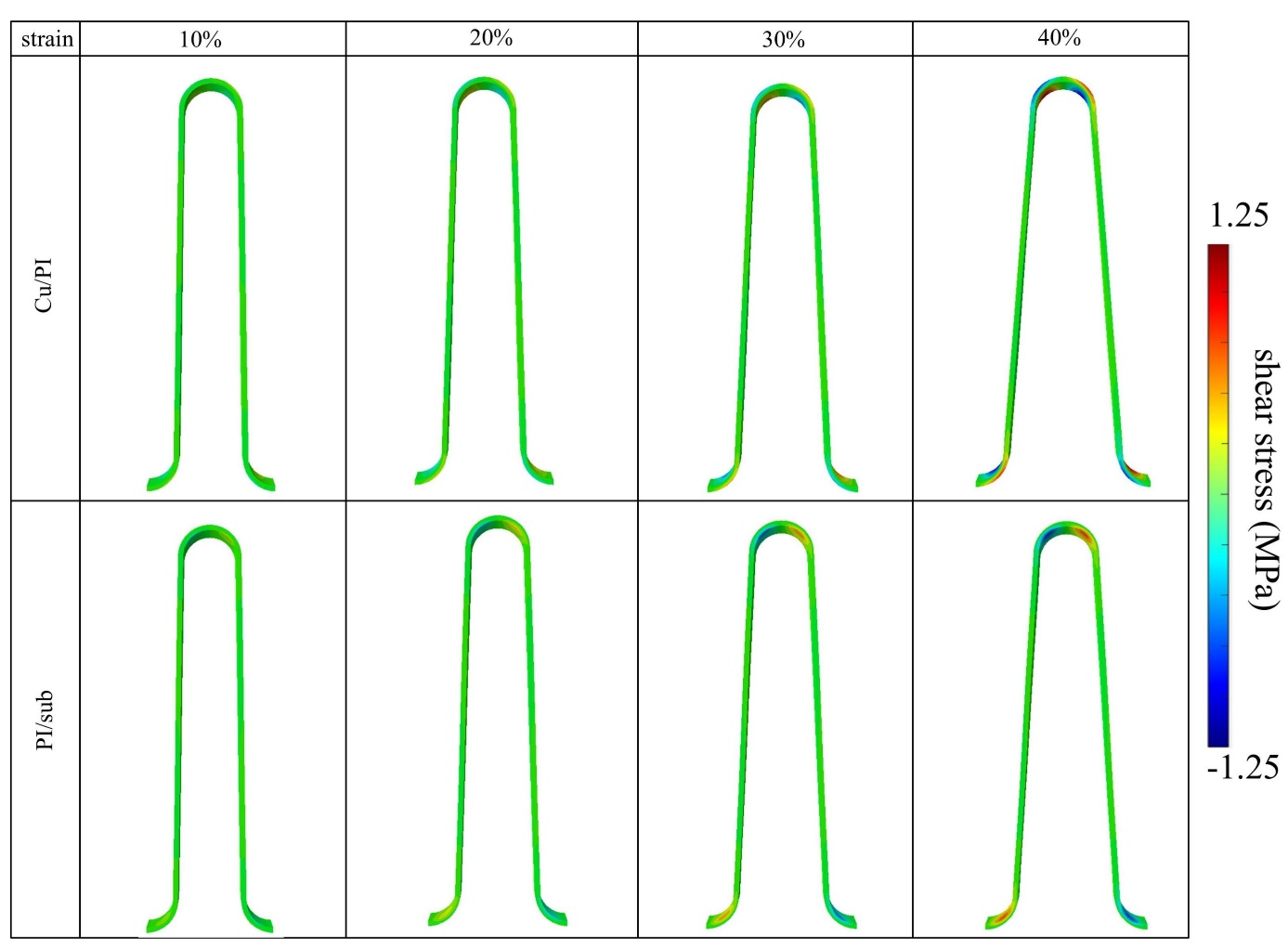


Fig. S16. the distribution of interfacial shear stress distribution at the PI/Cu interface and the PI/substrate interface.


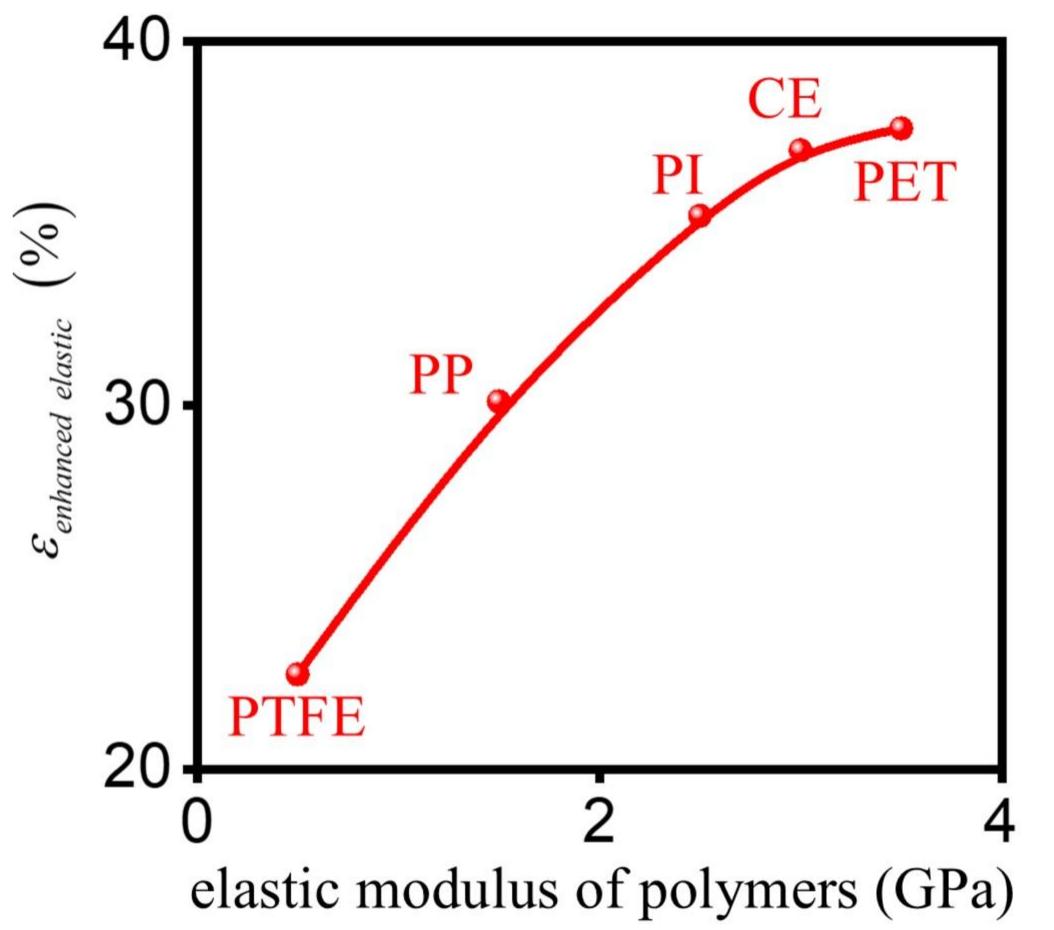


Fig. S17. Influence of the elastic modulus of polymer on the laminating strategy.


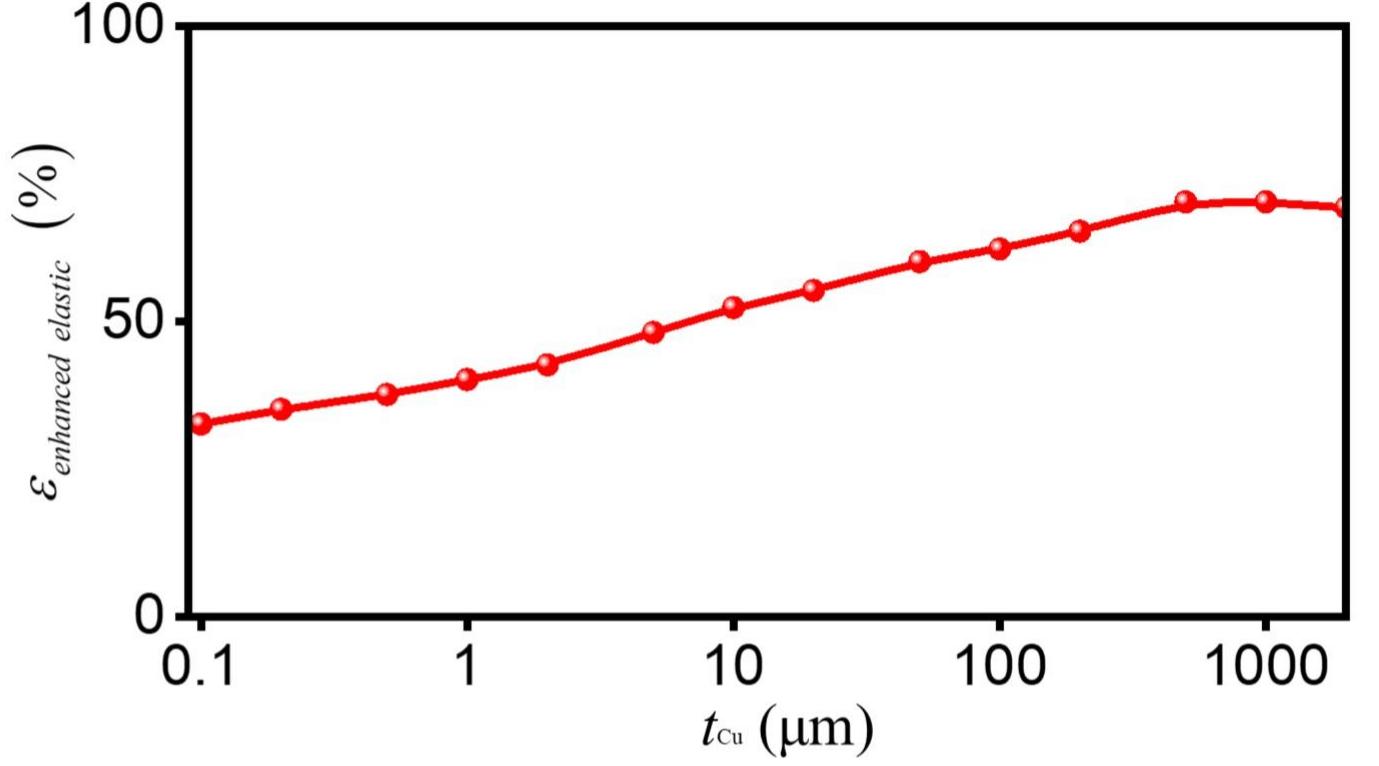


Fig. S18. Influence of the metal thickness on the laminating strategy.
